# Acetylation of Human TCF4 (TCF7L2) Proteins Attenuates Inhibition by the HBP1 Repressor and Induces a Conformational Change in the TCF4::DNA Complex

**DOI:** 10.1371/journal.pone.0061867

**Published:** 2013-04-15

**Authors:** Susanne Elfert, Andreas Weise, Katja Bruser, Martin L. Biniossek, Sabine Jägle, Niklas Senghaas, Andreas Hecht

**Affiliations:** 1 Institute of Molecular Medicine and Cell Research, Albert-Ludwigs-University Freiburg, Freiburg, Germany; 2 Faculty of Biology, Albert-Ludwigs-University Freiburg, Freiburg, Germany; 3 BIOSS Centre for Biological Signalling Studies, Albert-Ludwigs-University Freiburg, Freiburg, Germany; University of Colorado, Boulder, United States of America

## Abstract

The members of the TCF/LEF family of DNA-binding proteins are components of diverse gene regulatory networks. As nuclear effectors of Wnt/β-catenin signaling they act as assembly platforms for multimeric transcription complexes that either repress or activate gene expression. Previously, it was shown that several aspects of TCF/LEF protein function are regulated by post-translational modification. The association of TCF/LEF family members with acetyltransferases and deacetylases prompted us to investigate whether vertebrate TCF/LEF proteins are subject to acetylation. Through co-expression with p300 and CBP and subsequent analyses using mass spectrometry and immunodetection with anti-acetyl-lysine antibodies we show that TCF4 can be acetylated at lysine K_150_ by CBP. K_150_ acetylation is restricted to TCF4E splice variants and requires the simultaneous presence of β-catenin and the unique TCF4E C-terminus. To examine the functional consequences of K_150_ acetylation we substituted K_150_ with amino acids representing the non-acetylated and acetylated states. Reporter gene assays based on Wnt/β-catenin-responsive promoter regions did not indicate a general role of K_150_ acetylation in transactivation by TCF4E. However, in the presence of CBP, non-acetylatable TCF4E with a K_150_R substitution was more susceptible to inhibition by the HBP-1 repressor protein compared to wild-type TCF4E. Acetylation of K_150_ using a bacterial expression system or amino acid substitutions at K_150_ alter the electrophoretic properties of TCF4E::DNA complexes. This result suggests that K_150_ acetylation leads to a conformational change that may also represent the mechanism whereby acetylated TCF4E acquires resistance against HBP1. In summary, TCF4 not only recruits acetyltransferases but is also a substrate for these enzymes. The fact that acetylation affects only a subset of TCF4 splice variants and is mediated preferentially by CBP suggests that the conditional acetylation of TCF4E is a novel regulatory mechanism that diversifies the transcriptional output of Wnt/β-catenin signaling in response to changing intracellular signaling milieus.

## Introduction

Proper embryonic development and postnatal tissue homeostasis critically depend on precisely controlled gene expression. T-cell factors and lymphoid enhancer factor (TCF/LEF) constitute a family of highly conserved transcriptional regulators that is comprised of four members in humans: TCF1, LEF1, TCF3 and TCF4 (gene symbols *TCF7*, *LEF1*, *TCF7L1* and *TCF7L2*, respectively) [1], [2]. Loss-of-function studies in different organisms revealed that TCF/LEF proteins perform essential functions in various aspects of embryogenesis and adult life by acting as nuclear effectors of growth factor and mitogenic signaling cascades including the Wnt/β-catenin pathway [1]–[14].

Structural features common to all members of the TCF/LEF protein family include an N-terminal β-catenin-binding domain, interaction sites for Groucho-related-gene (Grg)/transducin-like enhancer of split (TLE) transcriptional corepressors, a high-mobility-group-box (HMG-box) DNA-binding domain and an adjacent nuclear localization signal (NLS) [1], [2]. Outside of these domains, however, the sequence similarity drops considerably. Structural divergence among TCF/LEF family members is further enhanced by alternative splicing. This divergence is most pronounced for the mouse and human *TCF7* and *TCF7L2* genes [15]–[18]. Thus, *TCF7L2* may give rise to more than one hundred transcript variants which can be grouped into three main categories of M-, S- and E-types based on their capacity to generate protein products (denominated TCF4) with structural similarities in their C-termini [15], [16], [18], [19]. TCF4E isoforms contain binding motifs for the carboxy-terminal binding protein (CtBP) [20], the lysine acetyl-transferase (KAT) p300 [21] and a so-called C-clamp, which represents a second DNA-binding domain in addition to the HMG-box [22], [23]. None of these features are found in the TCF4M and TCF4S variants. Differences in domain composition are likely to be the major cause of differences in promoter-specific transactivation potential between TCF4 protein isoforms and also within the TCF/LEF family [18], [21].

TCF/LEF proteins engage in transcriptional control in at least two different but not mutually exclusive ways. The HMG-box of TCF/LEF proteins belongs to the HMGB-domain subtype that recognizes and bends specific DNA sequences [24], [25]. Accordingly, TCF/LEF proteins can facilitate the juxtaposition of non-adjacent transcription factor binding sites by deformation of the DNA helix and thereby aid in the assembly of transcriptional complexes [24]. As an example of a second mode of action TCF/LEF proteins can occupy the promoters of Wnt/β-catenin target genes and support the recruitment of multi-component transcription complexes through direct interactions with specific protein-binding partners. Repressive complexes that form in the absence of a Wnt stimulus can contain Grg/TLE proteins, histone deacetylases (HDACs) and the carboxy-terminal binding protein (CtBP) [26]. Upon activation of the Wnt/β-catenin pathway, β-catenin translocates into the nucleus where it interacts with TCF/LEF proteins to displace or inactivate their co-repressors. Additionally, β-catenin-mediated recruitment of histone-modifying enzymes, chromatin remodelers and factors at the interface of the basal transcription machinery ultimately results in the activation of target gene transcription [26].

Interactions with their binding partners and other functions of TCF/LEF proteins are subject to regulation by diverse post-translational modifications. The Nemo-like kinase (NLK) phosphorylates human TCF4 at threonine residues T_178_ and T_189_; these modifications inhibit DNA-binding and promote TCF4 degradation [27], [28]. TCF4 is also phosphorylated by the TRAF2-and-NCK-interacting-protein-kinase (TNIK) at serine S_154_, which results in the activation of Wnt/β-catenin target gene expression through an unknown mechanism [29], [30]. Phosphorylation and sumoylation can also affect the intracellular distribution of TCF/LEF proteins [31]–[33].

Another modification that plays important roles in Wnt/β-catenin signaling is the reversible acetylation of lysine residues. HDACs that remove acetyl moieties and thereby antagonize KATs, compete with β-catenin for binding to TCF/LEF proteins [34]–[36] and are integral components of TCF/LEF-based transcriptional repression complexes [34]. Creb-binding protein (CBP) and the closely related p300 are two KATs that modify both histones and non-histone proteins [37], [38]. In *Drosophila melanogaster*, dCBP acts as transcriptional coactivator of Armadillo (the fly orthologue of β-catenin) [39]. Additionally, dCBP negatively regulates the interactions between Armadillo and LEF1 by acetylating lysine 25, which is located in the β-catenin binding domain of LEF1 [40]. Pop-1, a *C. elegans* TCF/LEF family member, can also be acetylated by CBP. In this case, however, acetylation affects the cytoplasmic/nuclear distribution of Pop-1 [41]. In vertebrates, the CBP-catalyzed acetylation of β-catenin increases its affinity for TCF4 [42], [43]. Furthermore, as in *Drosophila*, CBP and p300 function as crucial transcriptional cofactors of β-catenin [44]–[47]. However, depending upon the promoter-context, CBP and p300 may interchangeably function as coactivators or they can exhibit contrasting stimulatory and inhibitory activities, respectively [39], [48], [49].

HMG-box protein 1 (HBP1) is a sequence-specific DNA-binding protein and multifunctional transcriptional regulator involved in the coordination of differentiation and cellular proliferation in a large number of cellular backgrounds [50]–[58]. HBP1 has the properties of a tumor suppressor; this tumor suppressor activity is likely due to the ability of HBP1 to transcriptionally repress cell cycle regulators and the pro-inflammatory macrophage migration inhibitory factor [50], [51], [55], [58]. Moreover, HBP1 induces cellular senescence in response to oncogenic stress by upregulating CDKN2A/P16INK4A [54]. The expression and activity of HBP1 itself are under the control of oncogenic pathways involving miR-17-5p and different post-translational modifications [53], [59]. Additionally, a link between oncogenic Wnt/β-catenin signaling and HBP1 has been established by the finding that HBP1 physically interacts with two distinct domains in TCF4 to prevent its DNA binding and the resultant Wnt/β-catenin target gene expression in tumor cells [55]. HBP1 exerts a similar effect on c-MYC, a downstream target of Wnt/β-catenin signaling [52]. Thus, HBP1 is an important modulator and negative regulator of Wnt/β-catenin pathway activity. However, it is not known whether the inhibition of Wnt/β-catenin signaling by HBP1 occurs constitutively or can be regulated somehow.

While KATs and HDACs have been functionally linked to Wnt/β-catenin signaling and β-catenin/TCF-mediated transcriptional control, mechanistic insights and knowledge about the precise targets of these enzymes are still scarce. In particular, it would be interesting to know whether vertebrate TCF/LEF proteins not only participate in the recruitment of KATs and HDACs but are also subject to conditional acetylation themselves. Here, we show that CBP acetylates human TCF4 E-type splice variants at lysine residue K_150_. Acetylation of K_150_ requires β-catenin and the C-terminal tail of TCF4E2, raising the possibility that acetylation occurs in a triple protein complex. Although K_150_ is remote from the HMG-box and C-clamp DNA-binding domains of TCF4E isoforms, acetylation of K_150_ alters the mobility of TCF4::DNA complexes and changes their structure. Substitutions that mimic the acetylated or non-acetylated states of K_150_ have no impact on the transactivation capacity of TCF4E2 in general. However, they differentially affect the ability of the HBP1 repressor protein to attenuate TCF4E2 transcriptional activity. Thus, acetylation of K_150_ may have a regulatory function in context-dependent transcriptional control by TCF4 and protect against the inhibitory influence of HBP1.

## Results

### TCF4E2 is preferentially acetylated by CBP

**Figure 1:**
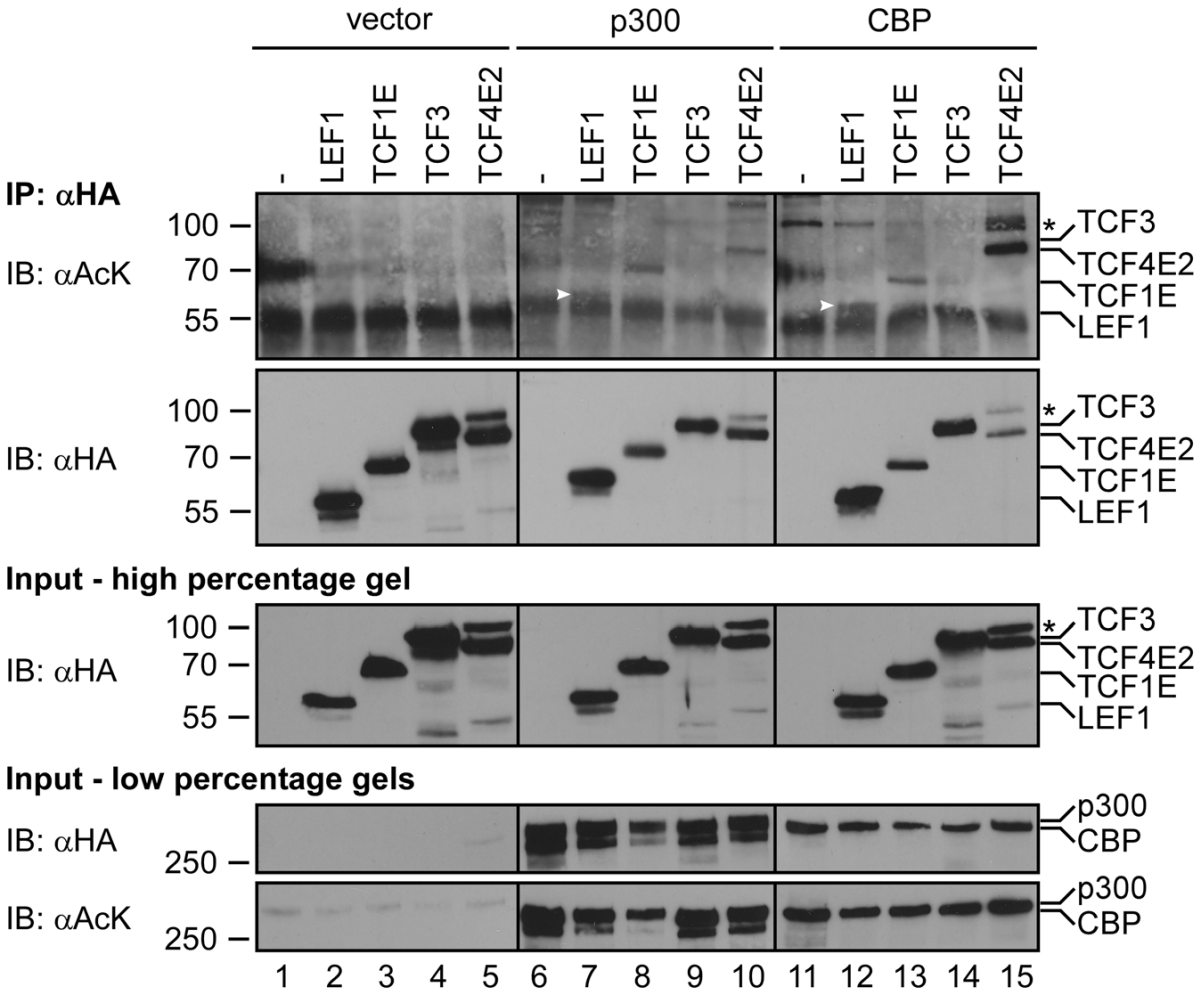
Acetylation of TCF/LEF family members by p300 and CBP. Expression vectors for HA-tagged versions of LEF1, TCF1E, TCF3 and TCF4E2 were transfected into HEK293 cells in the presence or absence of expression vectors for the acetyl-transferases p300 or CBP as indicated. To be able to detect p300 and CBP and to demonstrate comparable expression levels of the two proteins we used HA-tagged versions of p300 and CBP in this series of experiments. To detect acetylation of TCF/LEF proteins after immunoprecipitation with an antibody against the HA-tag, the samples were analyzed by SDS-PAGE and western blotting with a pan-anti-acetyl-lysine antibody (αAcK, top panel). The expression and immunoprecipitation of TCF/LEF proteins was controlled with the anti-HA antibody (middle panels). High percentage gels were used for the analyses of TCF/LEF family members. The expression of p300 and CBP in input material was also monitored by SDS-PAGE and western blotting with the anti-HA antibody. In addition, autoacetylation of p300 and CBP was detected with the pan-anti-acetyl-lysine antibody to demonstrate their enzymatic activity. Two distinct low percentage gels were used for the analyses of p300 and CBP expression and acetylation. The asterisks denote a species of TCF4E2 running at higher than expected molecular weight presumably due to an unknown post-translational modification. White arrowheads mark the location of LEF1. Molecular weight standards are shown on the left of the panels. IP: immunoprecipitation; IB: immunoblot.

To investigate the potential acetylation of different vertebrate TCF/LEF family members, LEF1, the TCF1E splice form, TCF3 and the TCF4E2 splice form were co-expressed with the acetyl-transferases p300 and CBP in HEK293 cells. All TCF/LEF factors harbored C-terminal HA-epitope tags that allowed for their immunoprecipitation from whole cell lysates. Immunoprecipitates were analyzed for the presence of acetylation by SDS-polyacrylamide gel electrophoresis (SDS-PAGE) and western blotting using a pan-acetyl-lysine antibody (Fig. 1, upper panel). For LEF1 and TCF1E we observed weak but clearly detectable acetylation at levels that were comparable in the presence of both p300 and CBP. TCF4E2 was also weakly acetylated by p300 but the signal was much more pronounced in the presence of CBP (Fig. 1, compare lanes 10 and 15). No acetyl-lysine immunoreactivity was observed for TCF3 neither upon co-expression with p300 nor with CBP. Comparable expression and similar efficiency of immunoprecipitation of the TCF/LEF family members was confirmed in the input material and immunoprecipitates (Fig. 1, middle panels). Moreover, p300 and CBP were expressed at similar levels and both proteins are enzymatically active as shown by their autoacetylation (Fig. 1, lower panels) [60], [61]. Thus, TCF/LEF family members appear to be differentially acetylated by p300 and CBP, with TCF4E2 being a preferential target of CBP.

### Acetylation of TCF4E2 at K_150_

Because acetylation of LEF1 was previously described [40] and we were intrigued by the differential effects of p300 and CBP, we focused on the acetylation of TCF4E2. To identify potential acetyl acceptor lysines, TCF4E2 was co-expressed with CBP, immunoprecipitated as before and then subjected to peptide analyses by mass spectrometry. Although TCF4E2 was not easily accessible by a typical proteomic approach because of its high proline content [62], nanoflow-HPLC-MS/MS (high performance liquid chromatography tandem mass spectrometry) measurement followed by Mascot search gave the first hint of acetylation at lysine residue K_150_ of TCF4E2 (Figures S1 and S2). The twofold charged parent ion of the TCF4E2-derived peptide DVQAGSLQSRQALK_150_ with K_150_ being acetylated was assigned a Mascot score value of 87 for the MS/MS spectrum and a mass deviation of 14 ppm for the parent ion (Figure S1). The total ion current signal was close to the detection limit. We additionally compared the MS/MS spectrum of the sample measurement with the spectrum of an acetylated synthetic reference peptide with the same amino acid sequence. Both showed good similarity. The threefold charged parent ion was also assigned by Mascot with a mass deviation of 7 ppm but a lower score value of 40 (Figure S2). The MS/MS spectrum also showed similarity to the reference peptide but contained a base peak which could not be assigned. Therefore, the evidence for acetylation of K_150_ is mainly supported by the spectrum of the twofold charged ion. The peptide was also found in its non-acetylated form in the sample suggesting that the TCF4E2 sample was only partially acetylated.

**Figure 2:**
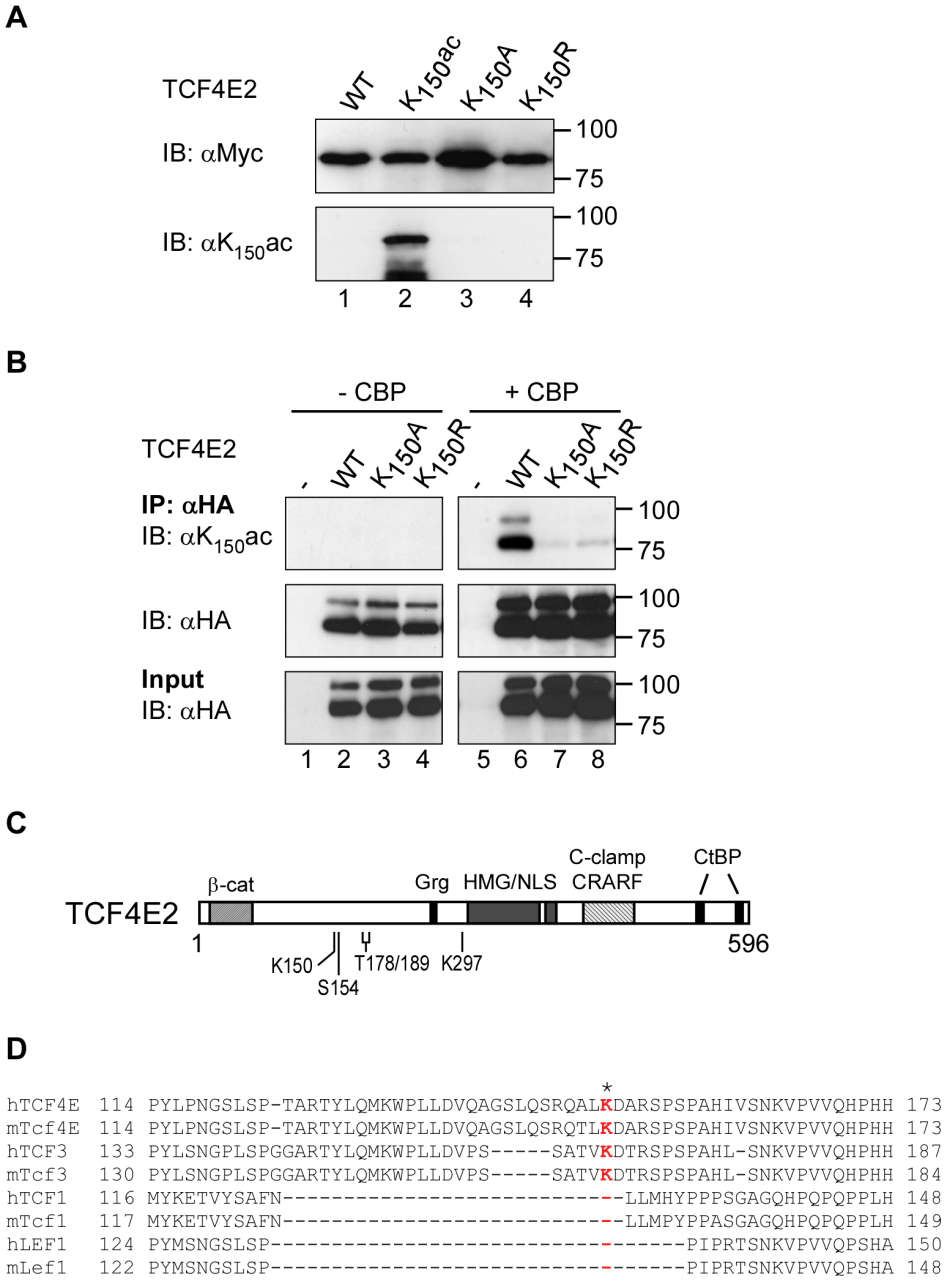
Acetylation of TCF4E2 at lysine 150. (**A**) Recombinant TCF4E2 variants expressed in *E. coli* were affinity-purified and analyzed by SDS-PAGE and western blotting with anti-c-Myc (top) and anti-K_150_ac antibodies (bottom). Molecular weight standards are indicated on the right. (**B**) HEK293 cells were transfected with expression vectors for HA-tagged TCF4E2 and TCF4E2 K_150_A or K_150_R mutants in the presence or absence of an expression vector for CBP (without HA-tag) as indicated. TCF4E2 was immunoprecipitated with anti-HA antibodies and precipitates were analyzed by SDS-PAGE and western blotting with anti-K_150_ac antibodies (upper panel) and anti-HA antibodies (middle panel). The presence of TCF4E2 proteins in the input material was also monitored by SDS-PAGE and western blotting using anti-HA antibodies (bottom panel). Molecular weight standards are shown on the right. (**C**) Schematic representation of TCF4E2 domain structure. β-Cat: β-catenin binding domain; Grg: Groucho/TLE binding motif; HMG: HMG-box; NLS: nuclear localization signal; CtBP: CtBP binding sites. Amino acid residues targeted by acetylation (K150), phosphorylation (S154, T178, T189) and sumoylation (K297) are marked. (**D**) Amino acid sequence conservation around TCF4E2 K_150_ (colored red and marked with an asterisk) in human (h) and mouse (m) TCF/LEF family members. The numbers reflect the positions of terminal amino acids shown for each of the factors. IP: immunoprecipitation; IB: immunoblot.

To independently confirm the acetylation of TCF4E2 at K_150_, a polyclonal peptide antibody against acetylated K_150_ (αK_150_ac) was produced. To test and verify the specificity of this peptide antibody, we generated bacterially expressed recombinant TCF4E2 proteins that harbored either lysine, alanine (TCF4E2K_150_A) or arginine (TCF4E2K_150_R) at amino acid position 150. In addition, we obtained TCF4E2 that was specifically acetylated at K_150_ (TCF4E2K_150_ac) by introducing a stop codon into the TCF4E2 cDNA at the position corresponding to K_150_ to exploit the site-specific acetylation system described by Neumann and coworkers [63]. All TCF4E2 variants were purified, separated by SDS-PAGE and analyzed by western blotting either with the K_150_ac antibody or with anti-c-Myc antibodies recognizing a C-terminal epitope tag common to all of them. While the anti-c-Myc antibodies detected all of the TCF4E2 variants, the K_150_ac antibody recognized only TCF4E2K_150_ac but not mutant or non-acetylated TCF4E2 (Fig. 2 A), which confirms the specificity of this antibody.

The αK_150_ac antibodies were then used to verify the acetylation of TCF4E2 at K_150_ in mammalian cells as indicated by the MS/MS analysis. TCF4E2, TCF4E2K_150_A and TCF4E2K_150_R were co-expressed with CBP in HEK293 cells, immunoprecipitated with anti-HA antibodies and analyzed by SDS-PAGE and western blotting (Fig. 2 B). In the absence of CBP, no acetylation could be detected. In the presence of CBP, a strong signal was detected for TCF4E2; this signal was significantly reduced for TCF4E2K_150_A and TCF4E2K_150_R (Fig. 2 B, upper panel), arguing that TCF4E2 is acetylated by CBP at K_150_.

Inspection of the location of K_150_ relative to the known domain structure of TCF/LEF family members and other protein features reveals that K_150_ resides within a part of TCF4E2 located between the β-catenin binding domain and the Grg/TLE interaction site (Fig. 2 C). Interestingly, potential phosphorylation sites for the serine/threonine kinases TNIK, homeodomain-interacting protein kinase 2 (HIPK2), and NLK [27], [30], [64] are in the near vicinity of K_150_, raising the possibility of cross-talk among these modifications. Amino acid sequence comparison within the TCF/LEF family and across different species shows that K_150_ is conserved in mouse and human TCF4E2 but not in mouse or human TCF1, LEF1, *Drosophila* dTCF or *C. elegans* POP1 (Fig. 2 D, data not shown). Mouse and human TCF3 proteins contain a stretch of amino acids related to the TCF4 sequence around K_150_ (Fig. 2 D). However, the absence of a GSLQS motif from TCF3 and other amino acid sequence deviations immediately preceding K_150_ may explain why TCF3, unlike TCF4, was not found to be acetylated in our initial analysis.

### A dual requirement of the β-catenin binding domain and C-terminal sequences for efficient acetylation of TCF4E splice variants at K_150_

**Figure 3:**
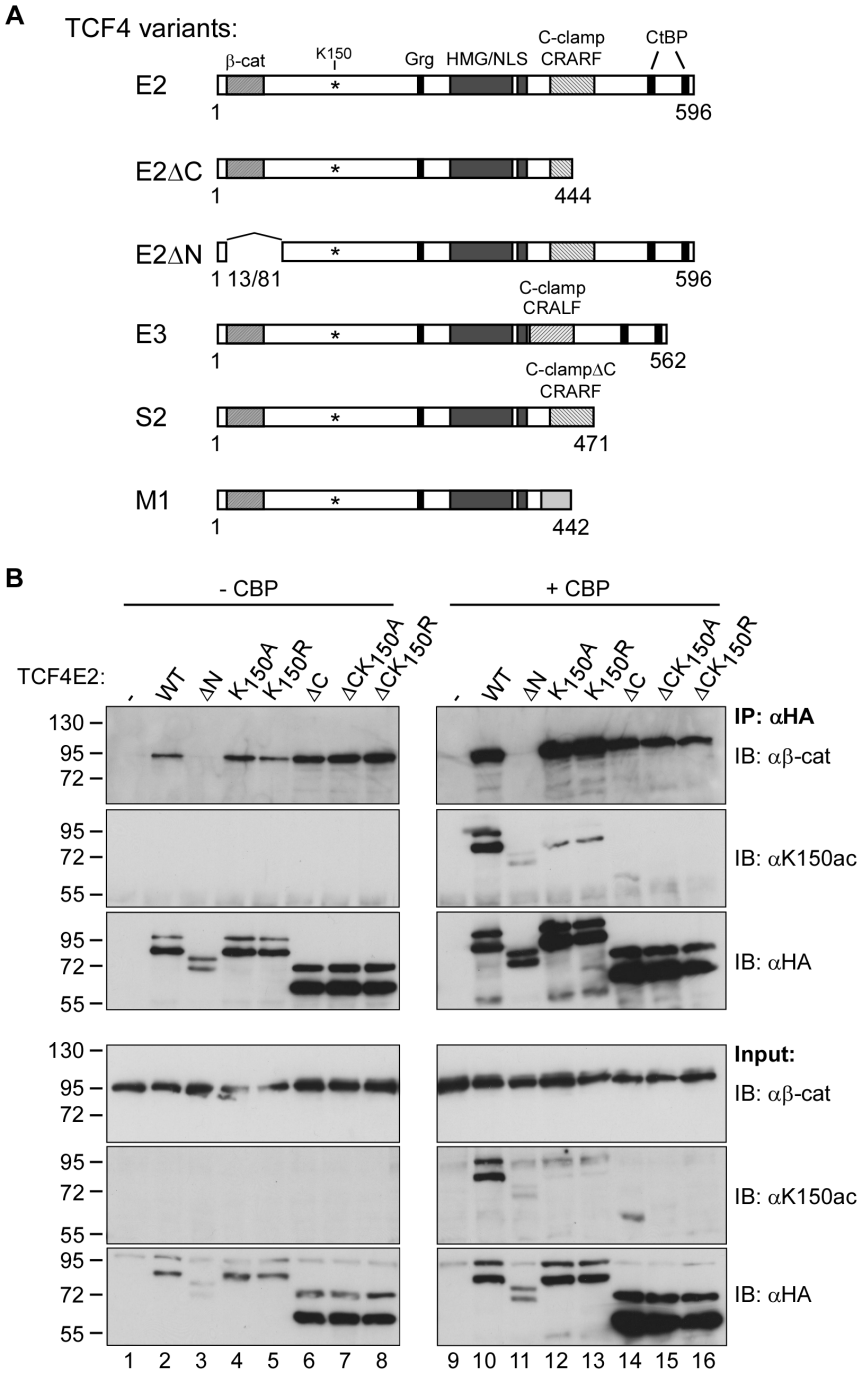
A dual requirement of the β-catenin binding domain and C-terminal sequences for efficient acetylation of TCF4E2 K_150_. (**A**) Schematic representation of protein structures of TCF4 splice variants and deletion mutants. β-Cat: β-catenin binding domain; Grg: Groucho/TLE binding motif; HMG: HMG-box; NLS: nuclear localization signal; CtBP: CtBP binding sites. CRARF/CRALF: amino acid signature motifs within the C-clamps of TCF4E2 and TCF4E3. K_150_ and coordinates of terminal amino acids are shown. (**B**) Expression constructs for TCF4E2, TCF4E2ΔN and TCF4E2ΔC (either wild-type or mutated at K_150_ to alanine or arginine as indicated) were transfected into HEK293 cells in the presence or absence of an expression vector for CBP. The TCF4E2 variants were immunoprecipitated from whole cell lysates with anti-HA antibodies. The presence of endogenous β-catenin in the precipitates or cell lysates was analyzed by SDS-PAGE and western blotting using anti-β-catenin antibodies (αβ-cat). The acetylation of TCF4E2 variants was analyzed by anti-K_150_ac antibodies (αK150ac) and the presence of TCF4E2 variants in the cell lysates and immunoprecipitates was analyzed by anti-HA antibodies (αHA). Molecular weight standards are indicated on the right. IP: immunoprecipitation; IB: immunoblot.

We and others have previously shown that p300 and CBP interact with β-catenin and that p300 binds to the C-terminal portion of TCF4E2 downstream of the HMG-box [21], [45]–[47]. To gain insight into the potential interplay of TCF4E2, CBP and β-catenin with respect to acetylation of K_150_, we performed co-immunoprecipitation experiments with TCF4E2, TCF4E2K_150_A, TCF4E2K_150_R and TCF4E2 deletion mutants lacking either the β-catenin binding domain (TCF4E2ΔN) or the C-terminus (TCF4E2ΔC) (Fig. 3 A). All TCF4E2 variants were present at comparable levels in whole cell extracts prepared from transfected HEK293 cells except for TCF4E2ΔN which was somewhat underrepresented (Fig. 3 B). This underrepresentation, however, was largely equalized in the immunoprecipitates. Furthermore, the presence of CBP led to a general increase in TCF4E2 levels. Regardless of these complications, the analyses of β-catenin co-immunoprecipitation and TCF4E2K_150_ acetylation yielded unequivocal results. All TCF4E2 variants except TCF4E2ΔN were able to precipitate β-catenin in the absence or presence of CBP (Fig. 3 B), suggesting that K_150_ and its acetylation do not impact on the interaction of TCF4E2 with β-catenin. In contrast, acetylation of TCF4E2, which was dependent on the co-expression of CBP (Fig. 3 B, compare lanes 2 and 10), was strongly reduced in the absence of β-catenin co-immunoprecipitation (Fig. 3 B, lane 11). The pronounced decrease of the acetylation signal seen with TCF4E2K_150_ mutants further confirms that K_150_ is targeted by CBP (Fig. 3 B, compare lanes 10, 12, 13). Deletion of the TCF4E2 C-terminus also impaired the CBP-mediated acetylation of K_150_, even though β-catenin still co-precipitated with TCF4E2ΔC (Fig. 3 B, lane 14). Thus, efficient acetylation of TCF4E2K_150_ shows a dual requirement for the N- and C-terminal protein sequences.

**Figure 4:**
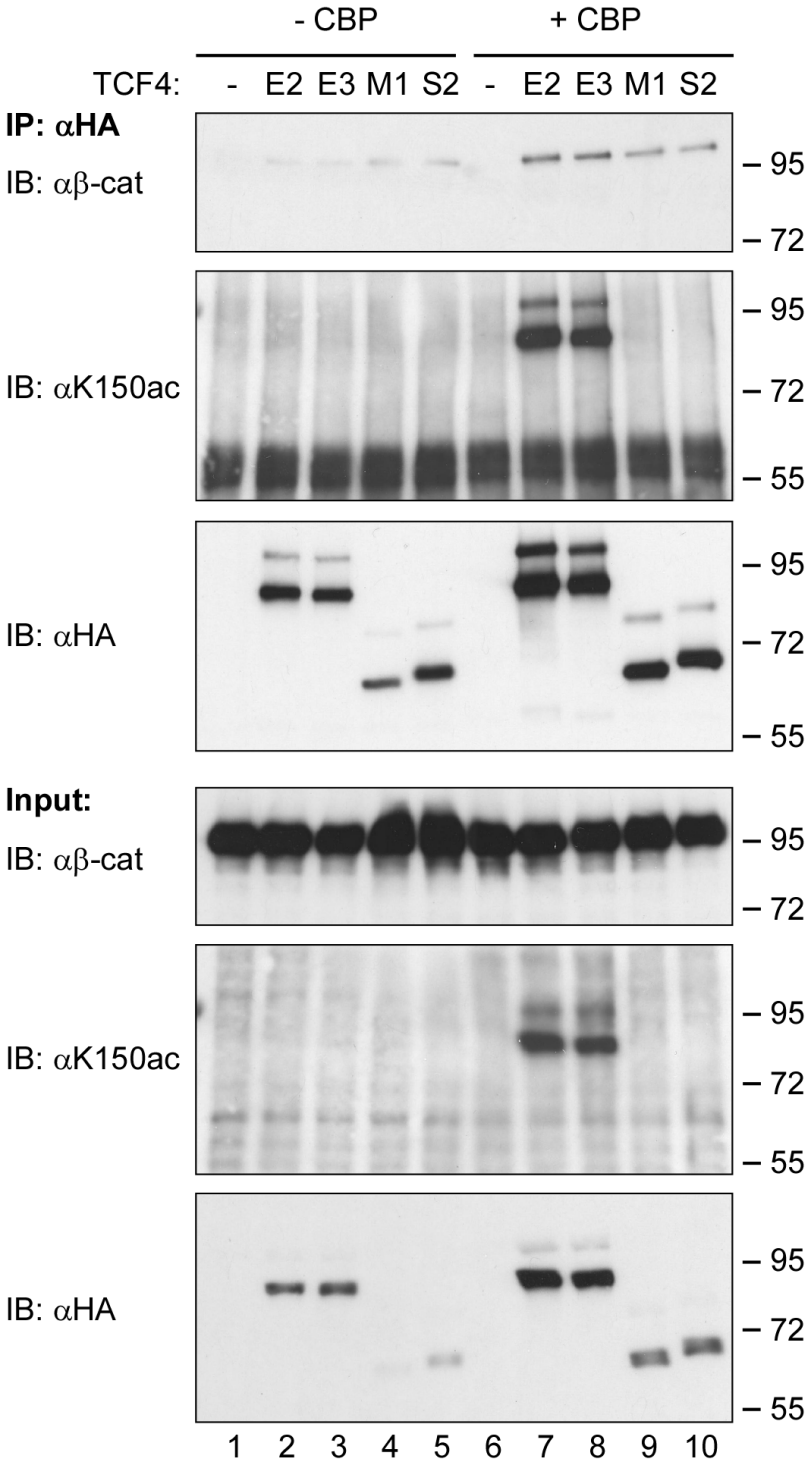
Acetylation of K_150_ is a special feature of TCF4E splice variants. Expression constructs for TCF4E2, TCF4E3, TCF4S2 and TCF4M1 were transfected into HEK293 cells in the presence or absence of an expression vector for CBP. TCF4 variants were immunoprecipitated from whole cell lysates with anti-HA antibodies. The presence of endogenous β-catenin in precipitates or cell lysates was analyzed by SDS-PAGE and western blotting using anti-β-catenin antibodies (αβ-cat). The acetylation of TCF4 isoforms was analyzed by anti-K_150_ac antibodies (αK150ac), and the presence of TCF4 isoforms in cell lysates and immunoprecipitates was analyzed by anti-HA antibodies (αHA). Molecular weight standards are indicated on the right. IP: immunoprecipitation; IB: immunoblot.

The finding that the C-terminus of TCF4E2 is required to promote the acetylation of K_150_ is intriguing because there are numerous naturally occurring TCF4 splice variants with C-terminal amino acid sequences that diverge from TCF4E2 to varying extents [15], [16], [18], [19], [65]. We therefore analyzed whether the TCF4E3 isoform and representatives of the M- and S-types of TCF4 splice variants (for structures see Fig. 3 A) also undergo acetylation at K_150_. TCF4E3 lacks amino acid residues derived from exon 13 and harbors a CRALF-type C-clamp. TCF4S2 has an incomplete C-clamp and resembles TCF4E2ΔC. The C-terminus of TCF4M1 differs completely and has none of the C-clamp elements at all. When tested in our combined co-expression/co-immunoprecipitation approach all TCF4 isoforms co-immunoprecipitated β-catenin (Fig. 4). However, only TCF4E2 and TCF4E3 were efficiently acetylated by CBP at K_150_. In contrast, neither TCF4S2 nor TCF4M1 exhibited K_150_ acetylation (Fig. 4), which is in agreement with the behavior of the TCF4E2ΔC deletion mutant. Thus, K_150_ acetylation appears to be a special feature of TCF4 E-type isoforms.

### K_150_ acetylation does not alter the transactivation capacity of TCF4E2

Next, we were interested in determining the potential impact of K_150_ and its acetylation on the protein function of TCF4E2. However, the acetylation of K_150_ does not appear to influence the intracellular localization of TCF4E2, nor does it affect TCF4E2 phosphorylation, ubiquitination or protein stability, as revealed by immunofluorescence analyses, phosphatase treatment and proteasome inhibitor studies (Figures S3 and S4). Finally, to determine whether acetylation at K_150_ modulates the transactivation capacity of TCF4E2, we performed reporter gene assays in HEK293 and U-2 OS cells and determined the luciferase activities driven by the β-catenin/TCF-inducible *Cdx1*, *Axin2* or *c-MYC* promoters (Figures S5 and S6). However, wild-type TCF4E2, and the TCF4E2K_150_A and TCF4E2K_150_R mutants that mimic the acetylated or non-acetylated states of K_150_, respectively, were indistinguishable in their ability to stimulate *Cdx1*, *Axin2* and *c-MYC* promoter activity upon co-expression with a constitutively active form of β-catenin in both cell lines. Thus, under the conditions of these experiments, K_150_ and its potential acetylation do not seem to affect the TCF4E2 transactivation capacity.

### K_150_ is involved in HBP1-mediated repression of TCF4E2 transactivation

**Figure 5:**
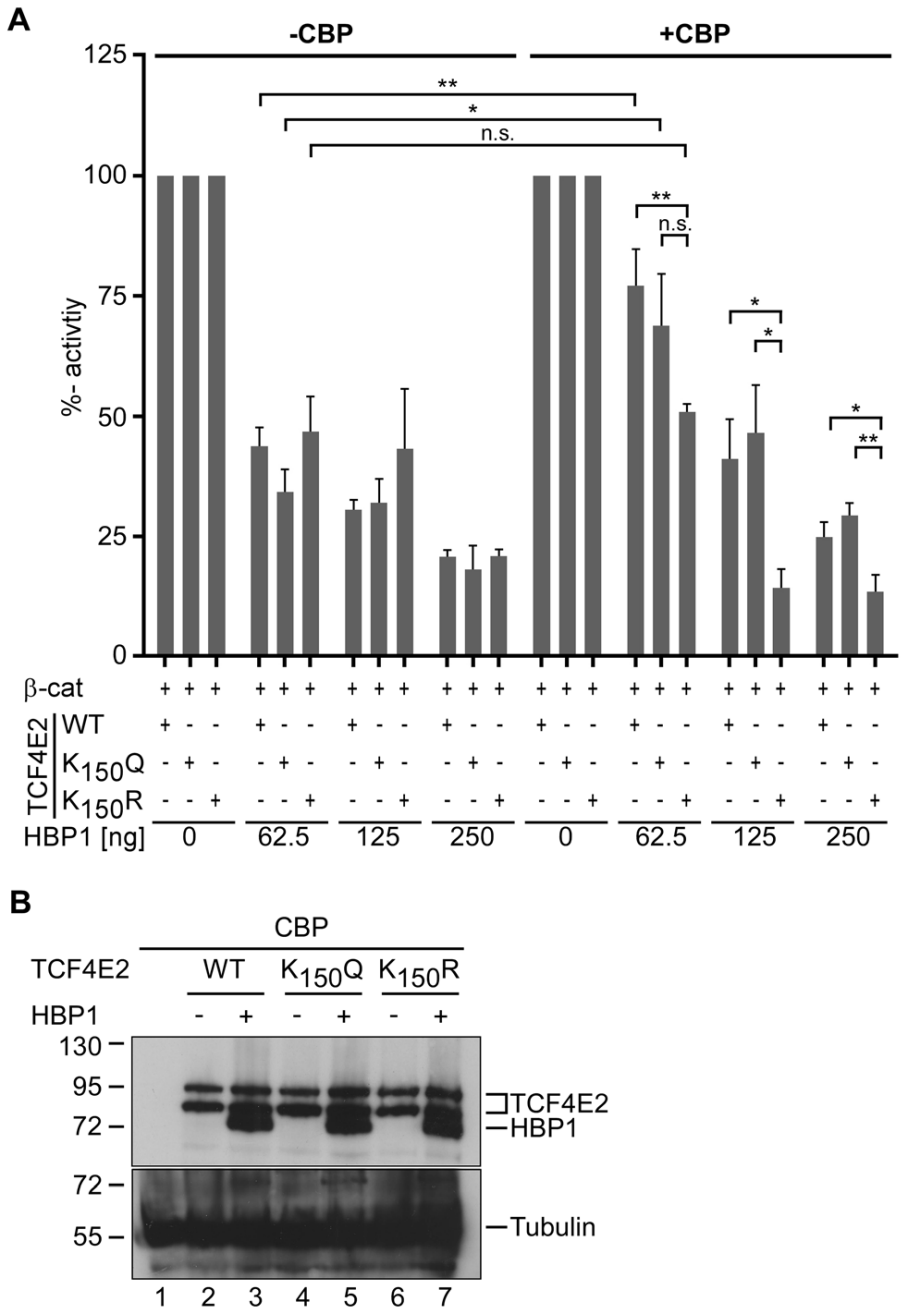
Mutation of TCF4E2 K_150_ attenuates the inhibitory influence of HBP1 in a CBP-dependent manner. (**A**) HEK293 cells were cotransfected with combinations of firefly and Renilla luciferase reporter genes and expression vectors for a constitutively active form of β-catenin, TCF4E2 variants, CBP and HBP1 as indicated. Firefly luciferase expression was driven by the promoter of the Wnt/β-catenin target gene *Cdx1*. Reporter gene activities were determined 40 h post transfection. Bars represent percentages of luciferase activity. Reporter activities obtained with TCF4E2 WT and the TCF4E2 K_150_ mutants in the absence of HBP1 were each set to 100 per cent both in the absence and presence of CBP. The average values and standard errors of the mean are shown. The asterisks mark statistically significant differences (**: p<0.01; *: p<0.05; n = 4; unpaired Student's t-test) (**B**) A relevant subset of the whole cell extracts used for luciferase measurements were also employed to monitor the expression levels of TCF4E2 variants and HBP1 by SDS-PAGE and western blotting with anti-HA antibodies. Note that the TCF4E2 variants and HBP1 are all marked by HA-epitope tags. The samples analyzed had been transfected with expression vectors for β-catenin, the TCF4E2 variants, CBP and the highest amount of HBP1 expression vector. Antibodies against α-tubulin were used to monitor equal loading. Molecular weight standards are shown on the left.

Even though the substitution of K_150_ did not alter the transcriptional activation by TCF4E2 *per se*, it is still possible that there are conditions in which K_150_ has an influence on the activity of TCF4E2. HBP1 is a transcriptional repressor that has been shown to interact with two domains in TCF4; these domains encompass amino acid residues 53–171 and 327–400 and thus include K_150_ and the TCF4 HMG-box, respectively [55]. HBP1 impairs transactivation by TCF4 by apparently preventing TCF4 DNA-binding. To test whether K_150_ plays a role in HBP1-mediated repression of TCF4 activity, we performed luciferase reporter gene assays in human HEK293 cells. Specifically, we asked whether the co-expression of HBP1 can affect the activation of a mouse *Cdx1* promoter construct by wild-type TCF4E2, TCF4E2K_150_Q, or TCF4E2K_150_R in the presence of constitutively active β-catenin. Additionally, CBP was co-expressed to force TCF4E2 acetylation. As expected, co-transfection of increasing amounts of HBP1 expression plasmid gradually decreased reporter gene activation by β-catenin and TCF4E2 (Fig. 5 A). In the absence of CBP, no significant difference between wild-type TCF4E2, TCF4E2K_150_Q, or TCF4E2K_150_R was observed. However, in the presence of CBP, wild-type TCF4E2 and TCF4E2K_150_Q proved to be less susceptible to inhibition by HBP1. Importantly, the protein levels of wild-type and mutant TCF4E2 were not reduced in the presence of HBP1 (Fig. 5 B). Overall, the observations that differences between wild-type TCF4E2, TCF4E2K_150_Q, and TCF4E2K_150_R arise only in the presence of CBP and that the TCF4E2K_150_Q mutant which mimics the acetylated state of K_150_, behaves like wild-type TCF4E2 suggest that non-acetylated TCF4E2 is the preferred target of HBP1.

In a first attempt to determine how K_150_ acetylation affects the inhibition of TCF4E2 by HBP1 we analyzed their interaction by co-immunoprecipitation. However, regardless of the presence or absence of CBP, HBP1 co-precipitated wild-type TCF4E2, TCF4E2K_150_Q, and TCF4E2K_150_R with similar efficiencies from whole cell extracts (data not shown). Apparently, the disruption of the TCF4::HBP1 complex does not underlie the protective effect of K_150_ acetylation.

### Acetylation of K_150_ alters DNA binding by TCF4E2

**Figure 6:**
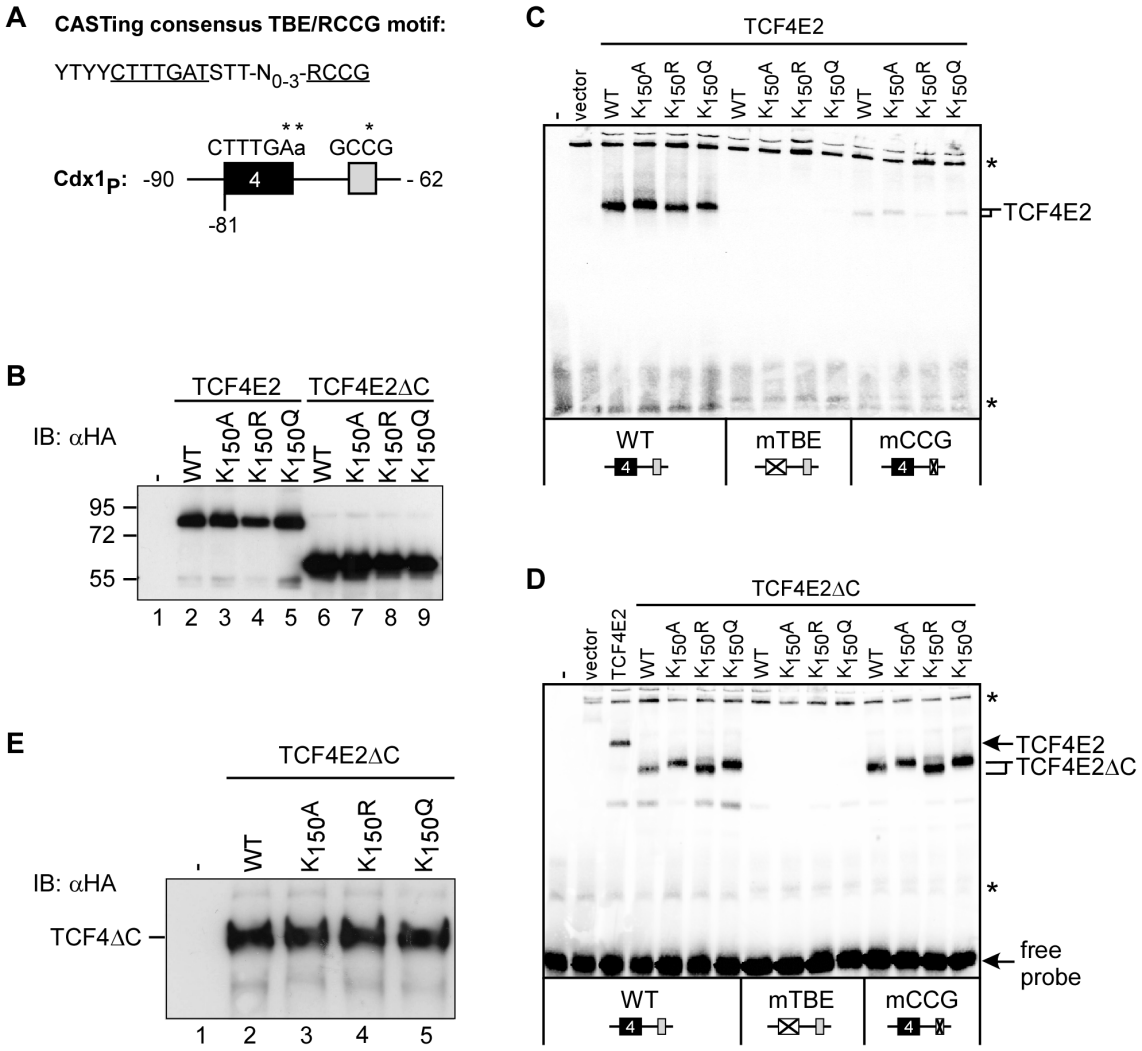
Mutations of K_150_ induce charge-independent changes in the migration of TCF4E2::DNA complexes *in vitro*. (**A**) Top: Extended consensus TCF-binding element (TBE) and adjacent 5′-RCCG-3′ motif derived from CASTing experiments. TBE core element and 5′-RCCG-3′ motif are underlined. Bottom: schematic depiction of the *Cdx1* TBE4 probe used for EMSAs. Numbers refer to coordinates of the *Cdx1* promoter (Cdx1_P_). Positions where mutations were inserted in the *Cdx1* TBE or 5′-RCCG-3′ motif are indicated by asterisks. (**B**) Wild-type and mutant TCF4E2 and TCF4E2ΔC proteins expressed *in vitro* were analyzed for equal abundance by SDS-PAGE and western blotting using anti-HA antibodies. Molecular weight standards are indicated on the left. Lane 1: control without protein (-). (**C, D**) EMSAs with TCF4E2 (C) or TCF4E2ΔC (D) using *Cdx1* TBE4 probes as depicted. Positions of protein::DNA complexes containing TCF4E2 and its derivatives are marked by arrows or brackets. Asterisks indicate non-specific bands. The control binding reaction received no protein (-) or wheat germ lysate mock-programmed with empty expression vector (vector). (**E**) Acidic urea PAGE of wild-type and mutant TCF4E2ΔC proteins expressed *in vitro*. TCF4E2ΔC variants were visualized upon electrophoresis by western blotting using anti-HA antibodies. Lane 1: control without protein (-).

HBP1 interacts with amino acid residues 53–171 of TCF4 and affects DNA binding by TCF4. By analogy, K_150_ and its acetylation may also have an impact on DNA binding by TCF4E2 and could thus indirectly interfere with HBP1 repressor function. To test this hypothesis, we performed electrophoretic mobility shift assays (EMSAs) with oligonucleotides containing *Cdx1* TCF-binding element (TBE) 4 and the adjacent 5′-RCCG-3′motif as probes (Fig. 6 A) [18]. First, we analyzed DNA binding by full-length TCF4E2, TCF4E2K_150_A, TCF4E2K_150_R and an additional TCF4E2K_150_Q mutant obtained by transcription and translation *in vitro*. TCF4E2K_150_Q was included in the assay because it shares structural similarity with acetylated K_150_. All TCF4E2 variants were present in similar amounts (Fig. 6 B) and bound to the wild-type *Cdx1* probe with equal efficiency (Fig. 6 C). As expected, mutation of the core TBE abolished the interaction with TCF4E2 proteins. Mutation of the 5′-RCCG-3′ motif also reduced the formation of TCF4E2::DNA complexes which is consistent with the idea that the 5′-RCCG-3′ motif provides an essential contact for the C-clamp specifically found in the TCF4E splice forms [18], [22], [23]. Interestingly, the protein::DNA complexes formed by TCF4E2 and TCF4E2K_150_R migrated slightly faster compared to those containing TCF4E2K_150_A and TCF4E2K_150_Q. The difference in mobility of the TCF4E2::DNA complexes was preserved with the probe containing a mutated 5′-RCCG-3′ motif (Fig. 6 C), suggesting that the effect of the amino acids at position 150 is independent of the C-clamp. To verify this hypothesis, we used TCF4E2 mutants lacking the C-clamp (TCF4E2ΔC) for the EMSA (Fig. 6 D). As with the full-length TCF4E2 forms, the shortened TCF4E2ΔC proteins were expressed at similar levels and bound the *Cdx1* TBE4 with equal efficiencies (Fig. 6 D). They also exhibit the same requirements for an intact TBE core motif. However, the absence of the C-clamp alleviates the necessity of the 5′-RCCG-3′ motif. Importantly, the wild-type and mutant TCF4E2ΔC::DNA complexes showed differences in mobility analogous to those observed with the full-length TCF4E2 variants but in an even more pronounced manner.

Differences in the mobility of the protein::DNA complexes formed by TCF4E2, TCF4E2K_150_A, TCF4E2K_150_R, and TCF4E2K_150_Q and the corresponding ΔC versions may arise from the presence or absence of a positively charged amino acid at position 150. To investigate this possibility, we used acidic urea PAGE to separate the TCF4E2ΔC variants according to their native charge. However, all TCF4E2ΔC variants migrate at the same position in acidic urea gels (Fig. 6 E). This result makes it highly unlikely that the retarded migration of the DNA complexes containing TCF4E2ΔCK_150_A and TCF4E2ΔCK_150_Q results from the lack of a single positively charged amino acid and instead argues in favor of differences in complex structure as the underlying cause.

**Figure 7:**
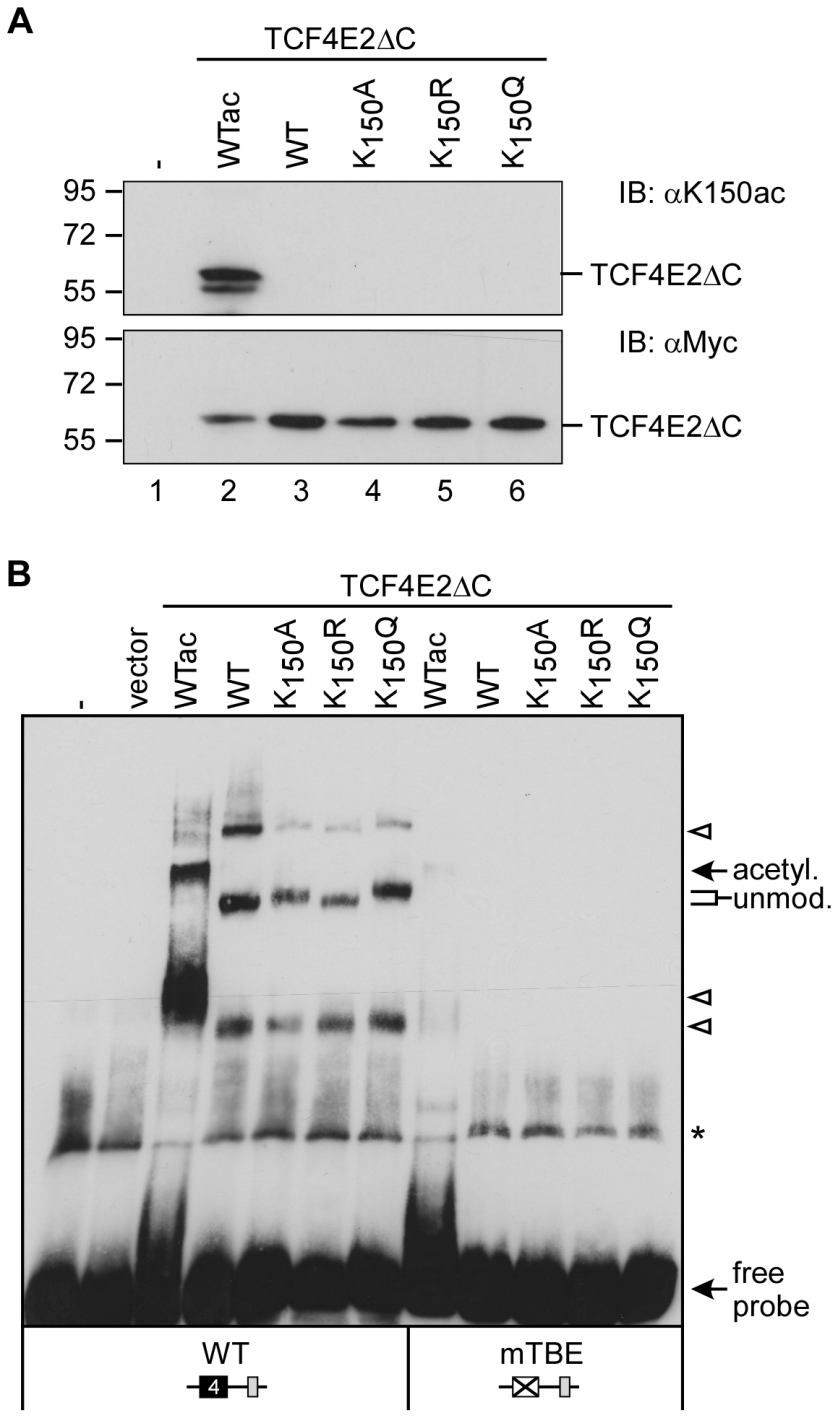
Acetylation of K_150_ affects the mobility of the TCF4E2::DNA complex more strongly than amino acid substitutions. (**A**) Recombinant TCF4E2ΔC variants expressed in *E. coli* were affinity-purified and analyzed by SDS-PAGE and western blotting with anti-K_150_ac (top) and anti-c-Myc antibodies (bottom). Molecular weight standards are indicated on the left. Lane 1: control without protein (-). (**B**) EMSA with TCF4E2ΔC using *Cdx1* TBE4 probes as depicted. Positions of protein::DNA complexes containing the unmodified wild-type and mutant TCF4E2ΔC, or the acetylated wild-type protein (WTac) are marked. Arrow-heads: TCF4::DNA complex generated by partially degraded TCF4 proteins or multimers. Asterisk: non-specific band. Control binding reaction received no protein (-) or mock protein purifications (vector) of bacteria transformed with empty expression vector.

TCF4E2 expressed *in vitro* is not acetylated (data not shown) and alanine, glutamine or arginine substitutions are only imperfect proxies for the acetylated and non-acetylated states of lysine residues, respectively. Therefore, the DNA binding properties of TCF4E2 when acetylated at K_150_ needed to be determined. For this determination, we used the bacterial expression system allowing site-specific acetylation of TCF4E2ΔC mentioned above [63]. TCF4E2ΔC, TCF4E2ΔCK_150_A, TCF4E2ΔCK_150_R, TCF4E2ΔCK_150_Q and TCF4E2ΔCK_150_ac were affinity purified from bacterial lysates and the presence of comparable protein amounts and K_150_ acetylation was determined by western blot (Fig. 7 A). When used in DNA-binding studies, the bacterial proteins showed a similar behavior to their counterparts expressed *in vitro*. As observed previously, DNA complexes formed by the non-acetylated wild-type protein and the K_150_R mutant migrated faster than those formed by the K_150_A and the K_150_Q variants. Strikingly, the DNA complex formed by acetylated TCF4E2ΔC migrated even more slowly (Fig. 7 B). No DNA binding was observed with a mutant *Cdx1* TBE4 or in the absence of bacterially expressed TCF4E2ΔC proteins. Taken together, the results of our DNA-binding studies suggest that acetylation of TCF4E2 at K_150_ has a strong influence on the structure of the TCF4E2::DNA complex.

## Discussion

Members of the TCF/LEF family of transcription factors perform critical functions as components of diverse gene regulatory networks in a variety of cell types and tissues [66]. Accordingly, their activities have to be tightly controlled. Previously, it was shown that the DNA binding, intracellular localization, and protein stability of TCF/LEF proteins can be regulated by phosphorylation and sumoylation [27]–[33], [64], [67]. The cytoplasmic-nuclear trafficking of invertebrate family members and the interaction with Armadillo, the fly orthologue of β-catenin, are controlled by lysine acetylation [40], [41]. Here, we have investigated whether vertebrate TCF/LEF proteins are also subject to acetylation. By co-expression with KATs and subsequent analyses using immunodetection with anti-acetyl-lysine antibodies we provide evidence that LEF1, TCF1E and TCF4E variants indeed can be acetylated. LEF1 and TCF1E are similarly modified by p300 and CBP whereas acetylation of TCF4E variants is much more pronounced in the presence of CBP. Acetylation of LEF1 within its β-catenin binding domain by *Drosophila* CBP had been previously reported [40] and was not further investigated. The acetylation of TCF1E and the TCF4E isoforms, however, is a novel finding and shows that TCF/LEF family members not only help to recruit KATs and HDACs to TCF/LEF target genes but also appear to be substrates for these enzymes themselves.

In many cases, CBP and p300 appear to be functionally interchangeable as coactivators of a large number of transcription factors but some differences between the two enzymes concerning their range of target genes exists [37], [38]. For example, both p300 and CBP cooperate with β-catenin in the regulation of certain Wnt target genes, but the two factors have opposing functions in Wnt/β-catenin mediated expression of the *BIRC5*/*Survivin* and *EPHB* receptor genes [48], [68], [69]. The molecular basis for these differences is unclear. Our finding that CBP can acetylate TCF4E isoforms much more efficiently than p300 further supports the idea that CBP and p300 can differentially contribute to Wnt/β-catenin signaling processes. It would also be interesting to explore whether differential acetylation of TCF4E plays a role in the contrasting activities of CBP and p300 at *BIRC5*/*Survivin* and similar genes.

Mass spectrometry provided first the evidence that K_150_ is an acetyl acceptor site in TCF4E2. This result was confirmed by mutagenesis and site specific anti-K_150_ac antibodies. Efficient acetylation of K_150_ required the simultaneous presence of the β-catenin binding site and the extended C-terminus of the TCF4E splice variants. This result is consistent with our previous identification of a p300/CBP interaction site at the C-terminus of TCF4E2 and raises the possibility that acetylation of TCF4E isoforms occurs in triple complexes containing TCF4E variants, CBP and β-catenin, although other scenarios cannot be excluded at this point. On the other hand, because of the involvement of β-catenin, one could hypothesize that K_150_ acetylation occurs conditionally and is regulated by Wnt signaling. However, this hypothesis awaits further investigation.

The *TCF7L2* gene, which codes for TCF4 proteins, generates a large number of structurally diverse splice variants [15], [16], [18], [19]. The TCF4M and TCF4S isoforms differ from TCF4E isoforms with respect to the amino acid composition of their C-termini. Generally, the transactivation capacity of the TCF4M and TCF4S isoforms is lower compared to TCF4E and they are likely to act upon different sets of target genes [18]. Here, we have shown that the TCF4M and TCF4S isoforms are not acetylated by CBP. This result further underscores the idea that TCF4 splice variants are functionally different and confirms that the C-terminus of TCF4 proteins is a critical determinant that confers unique properties upon TCF4E isoforms with respect to DNA binding, protein-protein interactions and post-translational modifications [18], [20]–[22]. Thus, the phenotypic effects of K_150_ acetylation on TCF4 splice variant function may be restricted to target genes that are specifically regulated by TCF4E isoforms.

K_150_ is located downstream of the β-catenin binding domain in TCF4 proteins and close to several serine and threonine residues that can be phosphorylated by TNIK, HIPK2 and NLK [27], [30], [64], [67]. These phosphorylation events affect transactivation by TCF4 proteins, DNA binding and protein stability. The location of K_150_ therefore raises the possibility of crosstalk between acetylation of K_150_ and phosphorylation of S_154_ and T_178/189_ in TCF4E variants. Corresponding interdependencies among these types of post-translational modifications have been reported for p53, histones and other factors [70], [71]. However, in our co-immunoprecipitation experiments, the acetylation of K_150_ or the mutation of this residue did not appear to influence the interaction with β-catenin. Thus, while complex formation with β-catenin is critical for K_150_ acetylation, there appears to be no reverse effect through positive or negative feedback. Similarly, our experiments with wild-type TCF4E2 and TCF4E2 mutants with alanine, arginine or glutamine substitutions at K_150_ involving phosphatase treatment and proteasome inhibition did not provide evidence that acetylation of K_150_ by CBP has an impact on TCF4E2 phosphorylation and turnover. Still, it may be premature to rigorously exclude the possibility of crosstalk between acetylation and phosphorylation of TCF4E isoforms. Overexpression of TCF4E2 and CBP may have biased our experimental system by exhausting the potentially limiting amounts of endogenous kinases in HEK293 cells. Future experiments involving TCF4 variants with additional substitutions at phospho-acceptor sites and co-expression of TNIK, NLK or HIPK2 should help to clarify whether there are any reciprocal effects between acetylation and phosphorylation of TCF4 proteins.

CBP has been shown to acetylate *C. elegans* Pop-1, altering intracellular distribution [41]. In our study, neither the co-expression of CBP nor mutation of K_150_ affected the intracellular localization of TCF4E2 which we found to reside exclusively in the nucleus. The apparent difference concerning changes in the localization of Pop-1 and TCF4E2 in response to CBP-mediated acetylation may be explained by the fact that CBP targets different protein domains in Pop-1 and TCF4E2 (this study and [41]). Furthermore, pairwise alignment of TCF4E2 and Pop-1 shows no or only very little amino acid sequence similarity in the regions corresponding to K_150_ in TCF4E2 and K_185,187,188_ in Pop-1. Thus, although acetylation affects several members of the TCF/LEF family, we hypothesize that this post-translational modification has been acquired independently during evolution of the TCF/LEF family to fulfill different regulatory purposes.

To date, our analyses concerning the acetylation of TCF4E2 have been performed under conditions of transient overexpression and with recombinant proteins *in vitro*. Therefore, it is an open question whether acetylation of TCF4E2 at K_150_ occurs under physiological conditions. Preliminary attempts to detect K_150_ acetylation after immunoprecipitation of TCF4 from colorectal cancer cells were not successful (A. W., S. E. and A.H., unpublished). We ascribe this result to the fact that K_150_ acetylation appears to be restricted to TCF4E isoforms and likely requires the formation of triple complexes between TCF4E isoforms, CBP and β-catenin. Currently available TCF4 antibodies and other analytical tools may not be suitable or sensitive enough to sufficiently enrich a subpopulation of TCF4E proteins that carry the K_150_ acetylation. The pronounced context-dependence of K_150_ acetylation also presents a challenge for the detection of gene regulatory phenotypes of TCF4E isoforms with amino acid substitutions at K_150_. Overcoming these hurdles may require the development of novel, isoform-specific TCF4 antibodies for selective immunoprecipitation and gene-replacement strategies to substitute K_150_ mutants for wild-type TCF4 proteins, thereby allowing the study of their functions at endogenous Wnt/β-catenin target genes.

When assessed in reporter gene assays using the *Cdx1*, *Axin2* and *c-MYC* promoters we found that the transactivation by TCF4E2 was neither impaired nor augmented by K_150_ mutation, suggesting that acetylation of K_150_ is not generally involved in transcriptional regulation by TCF4E variants. However, we cannot exclude that the functional importance of K_150_ and its acetylation presents differently depending upon experimental conditions, for example upon forced expression of NLK, TNIK, HIPK2 or when examined with other target gene promoters. Nonetheless, we favor the idea that acetylation of K_150_ exerts its effects only in a specific context. In agreement with this assumption we found that in the presence of CBP, wild-type TCF4E2 and TCF4E2K_150_Q which mimics acetylation, were partially protected against the inhibitory influence of HBP1. HBP1 is widely expressed and has a strong repressive effect on Wnt/β-catenin signaling [55], [58]. Therefore, it is easily conceivable that the interaction between HBP1 and TCF4E2 is subject to some form of regulation that could be provided by the acetylation of TCF4E2K_150_. Intriguingly, the two binding domains for HBP1 in TCF4E2 comprise amino acids 53–171 and 327–400, the latter corresponding to the HMG-box DNA binding domain [55]. Acetylation of TCF4E2 occurs at the more N-terminal of these domains and the phenotypic consequences of this modification manifest themselves in altered DNA binding of TCF4E2. This parallel and the observed functional consequences of the K_150_ mutation in reporter gene assays suggests to us that acetylation of TCF4E2 at K_150_ could be a relevant regulatory event to thwart the repressive influence by HBP1.

How could the acetylation of K_150_ in TCF4E2 interfere with HBP1-mediated repression? Mutation of K_150_ does not prevent the interaction of TCF4E2 with HBP1 in co-immunoprecipitation experiments (K. B. and A. H., unpublished) arguing that K_150_ acetylation does not directly interfere with the interaction of TCF4E2 and HBP1. Alternatively, the protective effect of K_150_ acetylation might manifest itself only under conditions in which HBP1 also exerts its inhibitory influence, namely DNA binding by TCF4E2. In support of this idea, we observed differences in the migratory behavior of protein::DNA complexes formed by wild-type and mutant TCF4E2. Migration of TCF4E2::DNA complexes was retarded by the substitution of K_150_ with alanine and glutamine and was slowed down even further upon acetylation of K_150_. We ruled out charge-dependent effects as an underlying cause because wild-type TCF4E2 and all mutant versions of TCF4E2 migrated equally fast in acid-urea gels. Moreover, the electrophoretic properties of TCF4E2K_150_A, TCF4E2K_150_Q and TCF4E2K_150_ac differed in EMSAs even though all three proteins show the same loss of a single positive charge. From this result, we conclude that charge neutralization and acetylation of K_150_ induce a structural change in the TCF4E2::DNA complex similar to what has been proposed for thymine DNA glycosylase [71]. Accordingly, the acetylation of K_150_ may result in a conformation of the TCF4E2::DNA complex that is at least partially resistant to the action of HBP1.

Conformational changes induced by acetylation may have additional implications aside from their potential impact on HBP1-mediated repression. TCF/LEF proteins are known to bend DNA and can function as architectural factors [24], [25]. Thus, one could speculate that different structures of TCF4E2::DNA complexes brought about by the acetylation of K_150_ contribute to the assembly or disassembly of multimeric transcription factor complexes at regulatory regions of genes regulated by TCF4E isoforms.

## Materials and Methods

### Plasmids and mutagenesis

Expression constructs for TCF1E, Lef1, TCF3, TCF4E2, TCF4E2ΔN, TCF4E2ΔC, p300, p300-HA, CBP, CBP-HA, HBP1, a constitutively active form of β-catenin and the luciferase reporter constructs pGL3b-Axin2, pGL3b-Cdx1 and pBV-luc c-MYC 4×TBE2 were described previously [21], [44], [55], [72]–[75]. Expression constructs for TCF4E3, TCF4S2 and TCF4M1 were derived from the TCF4E2 expression vector following a PCR-based cloning strategy as described [18]. Details are available upon request. The Renilla luciferase expression vector pRL-CMV was purchased from Promega, Heidelberg, Germany. TCF4E2 mutants TCF4E2 K_150_A, K_150_R and K_150_Q in pCS2+ [76] were generated by site-directed mutagenesis according to the Stratagene QuickChange mutagenesis protocol using the following primers: For K_150_A: 5′- GTAGACAAGCCCTCGCGGATGCCCGGTCC-3′; K_150_R: 5′-GTAGACAAGCCCTCAGGGATGCCCGGTC-3′; K_150_Q: 5′-GTAGACAAGCCCTCCAGGATGCCCGGTC-3′ (mutated codons are underlined). The TCF4E2 K_150_A, K_150_R and K_150_Q mutations were also introduced into the TCF4E2ΔC expression construct using the same protocol and primers. For site-specific acetylation, TCF4E2 K_150_TAG was generated in the pCal-c (Stratagene, Heidelberg, Germany) vector background with the same method using the primer 5′-GTAGACAAGCCCTCTAGGATGCCCGGTC-3′. All mutants were sequence verified by DNA sequencing using the BigDye® Terminator v1.1 cycle sequencing kit (Applied Biosystems, Darmstadt, Germany) and a MegaBACE 500 capillary sequencer (GE healthcare, Munich, Germany) operated by the sequencing core facility of the University Medical Center Freiburg. For bacterial expression, TCF4E2 coding sequences were transferred into the pCal-c expression vector using *Bam*HI*/Bst*BI restriction enzymes. Wild-type and mutant forms of TCF4E2ΔC were derived from these constructs by conventional cloning techniques (details available on request). To facilitate the detection of TCF4E2 proteins, constructs based on pCS2+ added a C-terminal HA-tag while constructs based on pCal-c added a C-terminal Myc-His-tag to TCF4E2 sequences.

### Cell culture, transient transfection and luciferase reporter assays

HEK293 and U-2 OS cells (ATCC # CRL-1573 and ATCC # HTB-96) were cultured in DMEM (PAN-biotech, Aidenbach, Germany) containing 10% fetal calf serum (PAN-Biotech, Aidenbach, Germany), 10 mM HEPES buffer (PAN-Biotech, Aidenbach, Germany), 1% MEM-non essential amino acids (Invitrogen, Karlsruhe, Germany) and 100 units/ml penicillin/streptomycin (Invitrogen, Karlsruhe, Germany). For western blotting and immunoprecipitations, 3×10^6^ cells in 10 cm culture dishes were transfected with 10 µg plasmid DNA for TCF/LEF proteins and 15 µg of expression vectors for p300 or CBP in 1 ml calcium-phosphate precipitate 6 hours after seeding. The DNA amounts were equalized with empty expression vector. For luciferase reporter assays, 1×10^5^ HEK293 cells were seeded per well of a 24-well plate. Cells were transfected with FuGENE6 reagent (Roche Applied Science, Mannheim, Germany) 4 h after plating. DNA mixtures of 100 ng of the pGL3b-Cdx1 firefly luciferase reporter, 10 ng of the Renilla luciferase expression vector pRL-CMV, 100 ng of plasmid DNA for expression of a constitutively active form of β-catenin, 50 ng of the expression constructs for WT and mutant TCF4E2, 250 ng of an expression vector for human CBP and three different amounts (62.5 ng, 125 ng or 250 ng) of an expression construct for rat HBP1 were used for the transfections. The total amounts of DNA transfected were kept constant by the addition of empty pCS2+ expression vector [76]. For the transfection experiments shown in Figures S5 and S6, 1×10^5^ HEK293 cells or 5×10^4^ U-2 OS cells seeded per well of a 24-well plate were transfected with FuGENE6 reagent (Roche Applied Science, Mannheim, Germany) 4 h after plating. DNA mixtures of 100 ng firefly luciferase reporter plasmids, 10 ng of the Renilla luciferase expression vector pRL-CMV, 100 ng of plasmid DNA for expression of a constitutively active form of β-catenin and three different amounts (75 ng, 150 ng or 300 ng) of each expression construct for the TCF4E2 variants were used for the transfections. The total amounts of DNA transfected were kept constant by addition of empty pCS2+ expression vector. Luciferase assays were performed 40 h after transfection as described [18], [21]. All results represent the average values of at least three independent experiments including standard deviations.

### Whole cell extracts and immunoprecipitation

To prevent enzymatic deacetylation, 1 µM trichostatin A (TSA) was added to the cells and to the cell lysis buffer 2 hours prior to protein preparation. Whole cell extracts were prepared 48 hours after transfection by lysing the cells in IPN_150_ buffer [50 mM Tris/HCl (pH 7.6), 150 mM NaCl, 5 mM MgCl_2_, 0.1% NP40, Complete™ protease inhibitor (Roche Applied Science, Mannheim, Germany), 1 µM TSA] for 30 minutes on ice. Cell lysates were cleared by centrifugation at 20000× *g* and 4°C for 10 min. The protein concentrations in the cell lysates were determined using the DC protein assay kit (BioRad, Munich Germany). For immunoprecipitation, whole cell lysate with a protein content of 1 mg was combined with 1 µg of anti-HA antibody (clone 3F10, Roche Applied Science, Mannheim, Germany) and 40 µl 50% (v/v) protein-G-sepharose (GE healthcare, Munich, Germany) and incubated over-night at 4°C under constant rotation. Afterwards, the probes were washed three times with IPN_150_ buffer and eluted by boiling in 2× SDS-loading buffer for 5 minutes at 95°C followed by SDS-PAGE analysis. For the purification of TCF4E2 for MS/MS analysis, HEK293 cells were transfected and lysed as described above. Cleared lysates from six 10 cm culture dishes were pooled and TCF4E2 was enriched by immunoprecipitation with 6 µg of anti-HA antibody (clone 3F10, Roche Applied Science, Mannheim, Germany) and 160 µl 50% (v/v) protein-G-sepharose (Roche Applied Science, Mannheim, Germany) in IPN_150_ over-night at 4°C. Following three washing steps with IPN_150_ lysis buffer, TCF4E2 was eluted from the protein-G-sepharose beads with 160 µl of 0.1 M glycine (pH 2.5) containing 1 µM TSA and Complete™ protease inhibitors (Roche Applied Science, Mannheim, Germany). After incubation at 4°C under gentle agitation the supernatant was removed from the protein-G-sepharose beads and neutralized by addition of 8 µl 1 M Na_2_HPO_4_ (pH 8.0). For further enrichment of the acetylated TCF4E2, a second immunoprecipitation step using a polyclonal rabbit pan-acetyl-lysine antibody (α-AcK; AB 3879, Millipore, Schwalbach, Germany) was performed. For this assay, the neutralized supernatant was added to 800 µl of 10 mM sodium phosphate buffer (pH 6.8) containing 2 µg of the anti-AcK antibody and 40 µl of 50% (v/v) protein-G-sepharose. After incubation for 3 h at 4°C, the protein-G-sepharose beads were washed three times with 10 mM sodium phosphate buffer (pH 6.8) and TCF4E2 was eluted with 50 µl 0.1 M TFA for 30 min at 4°C under gentle agitation. The supernatant was removed from the beads and neutralized by the addition of 2.5 µl of 1 M Na_2_HPO_4_ (pH 8.0). Fractions of the lysates and eluates from the different immunoprecipitation steps were used in western blots to monitor the presence and/or enrichment of non-acetylated or acetylated forms of TCF4E2.

### Silver staining of preparative gels and in gel endoproteinase digests

For preparative SDS-PAGE, subsequent silver staining and endoproteinase digests all the buffers and solutions were produced with HPLC grade water and all the equipment was thoroughly cleaned and treated with 5% formic acid prior to usage. In addition, all the steps were performed in a fume hood to prevent keratin contamination of the samples. After silver staining [77], TCF4E2 protein bands derived either from co-transfections with CBP expression construct or from transfections with TCF4E2 expression construct only were excised and used for in gel endoproteinase digests. As background controls for the MS/MS analysis, the gel pieces from empty neighboring lanes in the SDS-PAGE were excised and treated the same way as the TCF4E2 protein bands. Asp-N (Roche Applied Science, Mannheim, Germany) in gel endoproteinase digests were performed as follows: gel pieces were cut into small cubes with a scalpel and transferred to a 0.5 ml reaction tube. After the addition of 100 µl of 50 mM (NH_4_)HCO_3_, gel pieces were sonicated for 10 min in an ice water bath; this treatment was repeated for 20 min after the addition of 100 µl acetonitrile to the reaction. The supernatant was discarded and gel pieces were incubated for 10 min in water at room temperature. Supernatant was discarded following 10 min incubation at room temperature in 100 µl acetonitrile. The supernatant was removed and gel pieces were dried in a SpeedVac for 10 min. The dried gel pieces were incubated in 10 mM DTT in 50 mM (NH_4_)HCO_3_ for 30 min at 56°C in a thermo mixer at 500 rpm. The supernatant was removed, and gel pieces were incubated in 100 µl of 55 mM iodoacetamide in 50 mM (NH_4_)HCO_3_ for 30 min in the dark. Next, 100 µl of 50 mM (NH_4_)HCO_3_ was added, and after a 15 min incubation at room temperature, the supernatant was replaced with 100 µl of acetonitrile. After a 10 min incubation at room temperature, the supernatant was removed and gel pieces were dried in a SpeedVac as before. In each reaction tube 50 µl of sodium phosphate buffer (pH 8.0) containing 4 ng of Asp-N endoproteinase was added to the gel pieces following incubation at 37°C for 18 hours. The supernatant was removed and put into a new 0.5 ml reaction tube. The gel pieces were treated by another 15 min sonification step in 50 µl of acetonitrile, and the supernatant was added to the same new reaction tube. The supernatant was completely dried in a SpeedVac. Samples were kept at −20°C until MS/MS analysis.

### Nanoflow-HPLC- MS/MS analysis

Mass spectrometric measurements of protease-digested protein samples and synthetic control peptides were performed on a LTQ-FT mass spectrometer (Thermo Fisher Scientific, Bremen, Germany) coupled to an Ultimate3000 micro pump (Dionex, Idstein, Germany). Synthetic control peptides DVQAGSLQSRQALK and DVQAGSLQSRQALK(ac) were obtained from GenScript Corporation Piscataway, NJ, USA. HPLC-column tips (fused silica) with 75 µm inner diameter (New Objective Inc., Woburn, USA) were self packed [78] with Reprosil-Pur 120 ODS-3 (Dr. Maisch, Ammerbuch, Germany). For LCMS, peptide separation was performed using a gradient of 0.5% acetic acid (ACS Reagent, Sigma-Aldrich, Taufkirchen, Germany) in water to 0.5% acetic acid in 80% ACN (HPLC gradient grade, SDS, Peypin, France). Water and ACN were at least gradient-grade quality. The mass spectrometer was operated in the data-dependent mode and switched automatically between MS and MS/MS with a normalized collision energy setting of 35. Each MS scan was followed by a maximum of five MS/MS scans. The MASCOT-Software [79] (Matrixscience, London, United Kingdom) in combination with the NCBInr Database (National Center for Biotechnology Information, Bethesda, MD, USA) was used for protein identification (Search criteria: monoisotopic *m*/*z*, mass accuracy for MS: 15 ppm or better, for MS/MS 0.8 Da or better, up to three missed cleavages, variable modifications: Acetyl (K, N-term, Protein N-Term), Carbamidomethyl (C), Propionamide (C), Oxidation (M), Gln→pyro-Glu (N-term Q), Glu→pyro-Glu (N-term E).

### Western blotting and antibodies

For western blotting, proteins were transferred onto nitrocellulose membranes using a semi dry blotting apparatus after SDS-PAGE. As a blocking reagent, 2% skim milk powder in TBS-T [20 mM Tris/HCl (pH 7.6), 150 mM NaCl, 0.1% Tween-20] buffer was used. HBP1 and TCF4E2 were detected by a monoclonal rat anti-HA antibody (3F10, Roche Applied Science, Mannheim, Germany; 1∶2000). The acetylation of TCF/LEF proteins was analyzed with a polyclonal rabbit pan-acetyl-lysine antibody (α-AcK, AB 3879, Millipore, Schwalbach, Germany; 1∶1000). The acetylation of TCF4E2 K_150_ was detected with a polyclonal antibody to K_150_ac (Pineda Antibody Service, Berlin, Germany; 1∶500). This antibody was generated by immunizing rabbits with the acetylated peptide NH2-CRQALKacDAR-CONH_2_ derived from TCF4E2. Anti-K_150_ac specific polyclonal antibodies were purified from the resulting antisera by dual affinity purification. In the first step, a sepharose column charged with the modified peptide used for immunization was employed. From the eluate of this column antibodies reactive towards to non-acetylated TCF4E2 were removed through adsorption to a column charged with the unmodified peptide. Polyclonal rabbit antibodies were used to detect p300 and CBP (p300: C-20, Santa Cruz, Heidelberg, Germany; 1∶200; CBP: 06-294, Chemicon, Hofheim, Germany; 1∶1000). For detection of β-catenin and α-tubulin, mouse monoclonal antibodies (610154, BD Transduction Laboratories, Heidelberg, Germany; 1∶1000; T9026, Sigma-Aldrich, Taufkirchen, Germany; 1∶10000) were used. For the detection of recombinant His-purified TCF4E2 proteins expressed in *E. coli* a monoclonal mouse anti-c-Myc antibody (9E10, Roche Applied Science, Mannheim, Germany; 1∶2000) was used.

### Bacterial expression, site-directed acetylation and affinity-purification

For bacterial expression, TCF4E2 variants in the pCal-c expression vector were transformed into *E. coli* BL-21 and cultivated in LB-medium with ampicillin (150 µg/ml) to OD_600_ = 0.7–0.8 at 37°C. Protein expression was induced with 0.5 mM IPTG. After 3 to 5 hours of induction, cells were harvested by centrifugation at 10000× *g* for 10 minutes at 4°C and lysed in His-lysis buffer [50 mM NaH_2_PO_4_ (pH 8.0), 300 mM NaCl, 10 mM imidazole, 1 mg/ml lysozyme, 0.5% protease inhibitor cocktail for His-tagged proteins (Sigma-Aldrich, Taufkirchen, Germany)] for 30 minutes on ice followed by 6×10 seconds of sonication on ice. Lysates were cleared by centrifugation at 10000× *g* for 10 minutes at 4°C and stored on ice. For site-specific acetylation of TCF4E2 K_150_, the mutated expression vector containing the cDNA for TCF4E2 K_150_TAG was co-transformed into *E. coli* BL-21 together with pCDF_PylT-1 and pBK_AcRS3 encoding a tRNA_CUA_ and the according acetyl-lysyl-tRNA synthetase [63]. Cultures were grown in LB-medium supplemented with ampicillin (150 µg/ml), spectinomycin (50 µg/ml) and kanamycin (50 µg/ml) at 37°C. At OD_600_ = 0.7–0.8, 10 mM Nε-acetyl-lysine (Sigma-Aldrich, Taufkirchen, Germany) and 20 mM nicotinamide (Sigma-Aldrich, Taufkirchen, Germany) were added for additional 30 minutes before induction with 0.5 mM IPTG. After 5 hours cultivation, cells were collected by centrifugation at 10000 g for 10 minutes at 4°C and lysed as described above. For NiNTA purification, cleared lysates were incubated with NiNTA-Agarose (Qiagen, Hilden, Germany) over-night at 4°C with constant rotation. The agarose was collected by centrifugation at 600× *g* for 3 minutes and the unbound fraction was removed. The agarose was washed twice with His-wash buffer [50 mM NaH_2_PO_4_ (pH 8.0), 300 mM NaCl, 10 mM imidazole] for 10 minutes followed by centrifugation at 600 g for 3 minutes. After the second washing step, the agarose was resuspended in 1 bed volume of His-wash buffer and applied to a chromatography column. 10 bed volumes His-wash buffer were added to the column for the third washing step. His-tagged proteins were eluted with His-elution buffer [50 mM NaH_2_PO_4_ (pH 8.0), 300 mM NaCl, 250 mM Imidazol, 0.5% protease inhibitor cocktail for His-tagged proteins (Sigma-Aldrich, Taufkirchen, Germany)], and 1 ml fractions were collected and analyzed by SDS-PAGE. Elution fractions that contained the highest amounts of the His-tagged protein were pooled, dialyzed twice against 20 mM HEPES (pH 7.9), 75 mM NaCl, 2 mM MgCl_2_, 10% glycerol over-night at 4°C, concentrated 10-fold using Amicon Ultra centrifugal units (Millipore, Schwalbach, Germany) and stored at −80°C.

### Immunofluorescence

For indirect immunofluorescence, 1×10^5^ U-2 OS cells seeded onto glass cover slips in 24-well plates were transfected with 100 ng of plasmid DNA using FuGENE6 (Roche Applied Science, Mannheim, Germany) according to the recommendations of the manufacturer. After 48 hours, the cells were fixed with 4% paraformaldehyde, permeabilised with 0.5% Triton X-100 and stained with anti-HA antibody (3F10, Roche Applied Science, Mannheim, Germany; 1∶200) over-night. As a secondary antibody, goat anti-rat Alexa-555 was used at a 1∶200 dilution (Molecular Probes, Darmstadt, Germany). Nuclei were counterstained with DAPI (1∶1000).

### Treatment with λ-phosphatase and proteasome inhibitor

To determine the phosphorylation status of proteins, 20 µg of whole cell lysate was treated with 400 units of λ-phosphatase (NEB, Frankfurt, Germany) for 20 minutes at 30°C and analyzed by SDS-PAGE and western blotting. For analysis of proteasomal degradation, cells were treated with 20 µM MG132 in DMSO or a solvent control for two hours prior to cell lysis. Whole cell lysates were prepared as described above and further analyzed by SDS-PAGE and western blotting.

### Transcription and translation *in vitro* and electrophoretic mobility shift assays (EMSAs)

Proteins for use in DNA binding assays were either expressed in *E. coli* and purified as described above, or they were produced by transcription and translation *in vitro* using the TNT SP6 high-yield wheat germ protein expression system (Promega, Heidelberg, Germany) with 6 µg of plasmid DNA in 50-µl reactions. Equal expression levels of the different TCF4E2 variants obtained either way were controlled by western blotting. Binding reactions for EMSAs including biotinylated oligonucleotides representing *Cdx1* TCF-binding element 4 (TBE4) were composed as before and the samples were further processed as described [18].

### Acidic Urea PAGE

To determine if there are any potential influences of protein charge on the migratory behavior of TCF4E2, material transcribed and translated *in vitro* was separated by acidic urea PAGE [3M urea, 5% acetic acid, 10% polyacrylamide∶bisacrylamide] and blotted towards the cathode onto a PVDF membrane in 5% acetic acid using a semi-dry blotting apparatus. After blotting, the membranes were equilibrated in TBS-T for at least 20 minutes and blocked with 2% skim milk in TBS-T buffer. Proteins were detected with a monoclonal rat anti-HA antibody (3F10, Roche Applied Sciences, Mannheim, Germany; 1∶2000).

## Supporting Information

Figure S1 **Evidence for acetylation of TCF4 at K_150_.** Comparison of the MS/MS spectra for peptides found in the AspN-digested TCF4 sample and assigned by MASCOT (**A, C**) versus the MS/MS spectra of acetylated and non-acetylated versions of the synthetic peptide **DVQAGSLQSRQALK (B, D)**. The pictures show the MS/MS spectra of the doubly charged precursor ions as assigned by Mascot. (**A**) Sample spectrum assigned to the peptide acetylated at the lysine with a Mascot score value of 87. (**B**) Spectrum of acetylated synthetic control peptide. (**C**) Sample spectrum assigned to the non-acetylated peptide with a Mascot score value of 65. (**D**) Spectrum of non-acetylated synthetic control peptide.
(TIF)

Figure S2 **Evidence for acetylation of TCF4 at K_150_.** Comparison of the MS/MS spectra for peptides found in the AspN-digested TCF4 sample and assigned by MASCOT (**A, C**) versus the MS/MS spectra of acetylated and non-acetylated versions of the synthetic peptide **DVQAGSLQSRQALK (B, D)**. The pictures show the MS/MS spectra of the triply charged precursor ions as assigned by Mascot. (**A**) Sample spectrum assigned to the peptide acetylated at the lysine with a Mascot score value of 40. (**B**) Spectrum of acetylated synthetic control peptide. (**C**) Sample spectrum assigned to the non-acetylated peptide with a Mascot score value of 26, which is below the Mascot threshold value for indication of identity at p<0.05. (**D**) Spectrum of non-acetylated synthetic control peptide.
(TIF)

Figure S3 **Intracellular localization of TCF4E2 WT, K_150_A and K_150_R.** U-2 OS cells transfected with expression constructs for the TCF4E2 variants with or without CBP were stained with anti-HA antibodies and secondary antibodies coupled to Alexa-555 to visualize the TCF4E2 variants (TCF4, red). Nuclei were counterstained with DAPI. Phase contrast and overlay of TCF4E2 and DAPI staining are shown. Bar: 20 µm.
(TIF)

Figure S4 **Acetylation of K_150_ does not affect phosphorylation or proteasomal degradation of TCF4E2.** (**A**) To analyze if K_150_ acetylation influences the phosphorylation of TCF4E2, extracts of HEK293 cells transfected with the expression constructs for TCF4E2, TCF4E2K_150_A or TCF4E2K_150_R in the absence or presence of CBP were treated with λ-phosphatase (λ-PPase) and analyzed by SDS-PAGE and western blotting with anti-HA antibodies. Prior to λ-phosphatase treatment, all TCF4 variants showed comparable migration patterns by SDS-PAGE, suggesting that they are equally phosphorylated. λ-phosphatase treatment resulted in a mobility shift and faster migration of the TCF4 variants due to dephosphorylation. Again, wild-type TCF4E2 and TCF4E2 mutants showed uniform behavior. (**B**) HEK293 cells transfected with expression constructs for TCF4E2, TCF4E2K_150_A or TCF4E2K_150_R with or without CBP were treated with 20 µM MG132 for two hours prior to cell lysis and whole cell extracts were analyzed by SDS-PAGE and western blotting with anti-HA and anti-β-catenin (αβ-cat) antibodies. For TCF4E2 no change in protein amount and no additional protein bands that would indicate polyubiquitination were detected. The presence or absence of CBP made no difference. In contrast, for β-catenin, MG132 treatment resulted in the appearance of additional protein bands and a stronger signal, suggesting polyubiquitination of the protein. Molecular weight standards are indicated on the right of the panels.
(TIF)

Figure S5 **Mutation of K_150_ has no influence on the transactivation capacity of TCF4E2 at different promoters in HEK293 cells.** HEK293 cells were cotransfected with combinations of firefly and Renilla luciferase reporter genes, control vector, expression vector for a constitutively active form of β-catenin and increasing amounts of TCF4E2 variants as indicated. Firefly expression was driven by promoters from the Wnt/β-catenin target genes *Cdx1* (**A**), *Axin2* (**B**) and *c-MYC* (**C**). Reporter gene activities were determined 40 h post transfection. Bars represent relative luciferase activity compared to values obtained with the lowest amount of TCF4E2 WT expression vector and β-catenin (set to 100%). The average values and standard deviations from at least three independent experiments are shown. (**D**) Expression levels of all TCF4E2 variants in whole cell extracts of cells transfected with 150 ng DNA as used in the luciferase reporter assays was controlled by SDS-PAGE and western blotting using anti-HA antibodies. Molecular weight standards are shown on the left.
(TIF)

Figure S6 **Mutation of K_150_ has no influence on the transactivation capacity of TCF4E2 at different promoters in U-2 OS cells.** U-2 OS cells were cotransfected with combinations of firefly and Renilla luciferase reporter genes, control vector, expression vector for a constitutively active form of β-catenin and increasing amounts of TCF4E2 variants as indicated. Firefly expression was driven by promoters from the Wnt/β-catenin target genes *Cdx1* (**A**), *Axin2* (**B**) and *c-MYC* (**C**). Reporter gene activities were determined 40 h post transfection. Bars represent relative luciferase activity compared to values obtained with the lowest amount of TCF4E2 WT expression vector and β-catenin (set to 100%). The average values and standard deviations from at least three independent experiments are shown.
(TIF)
