# Supplementary figures and images for: Acetylation of Human TCF4 (TCF7L2) Proteins Attenuates Inhibition by the HBP1 Repressor and Induces a Conformational Change in the TCF4::DNA Complex
Source: PLoS One. 2013 Apr 15;8(4):e61867. doi: 10.1371/journal.pone.0061867 (PMC3626699; doi:10.1371/journal.pone.0061867)

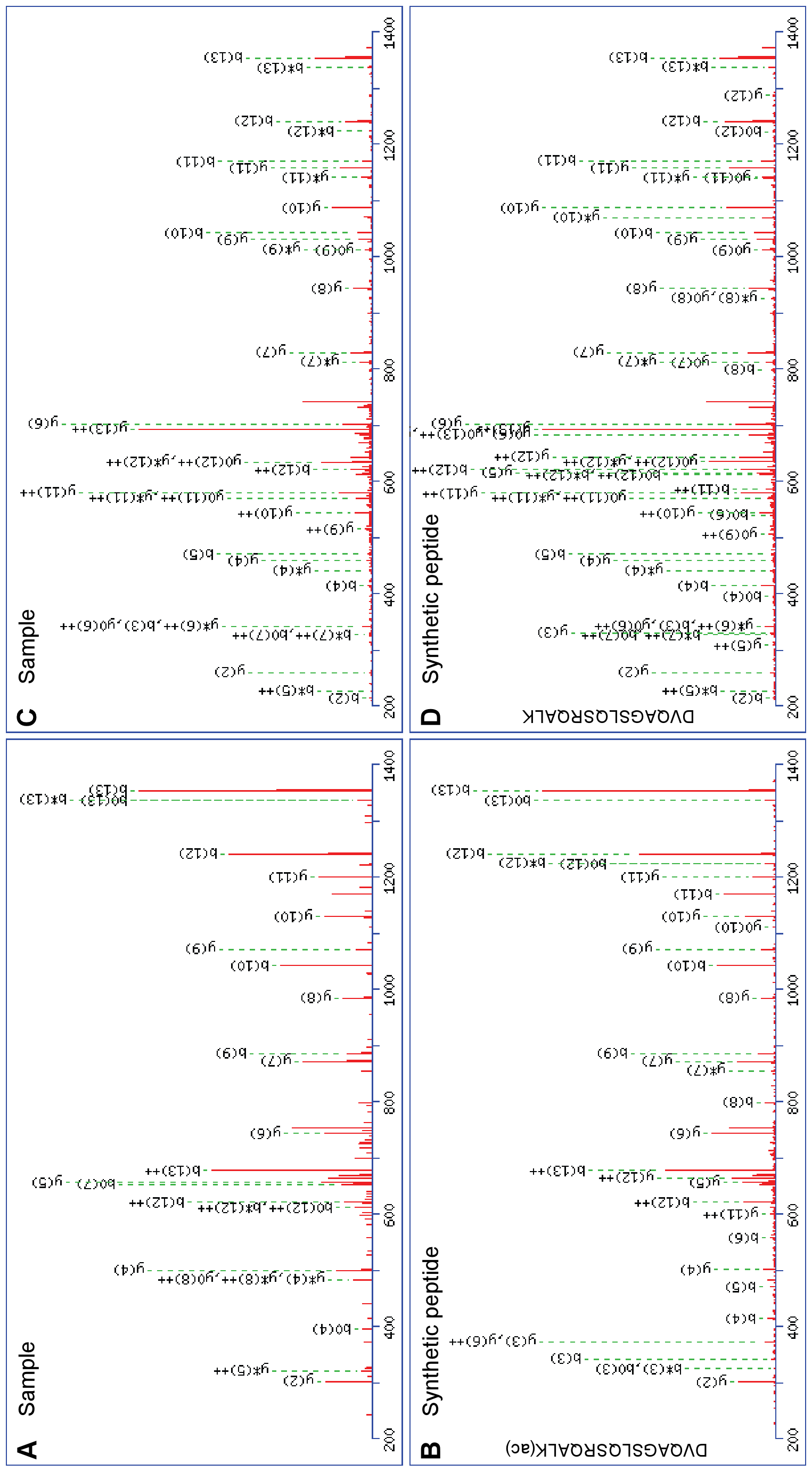

Supplement: Figure S1 — Evidence for acetylation of TCF4 at K150. Comparison of the MS/MS spectra for peptides found in the AspN-digested TCF4 sample and assigned by MASCOT (A, C) versus the MS/MS spectra of acetylated and non-acetylated versions of the synthetic peptide DVQAGSLQSRQALK (B, D). The pictures show the MS/MS spectra of the doubly charged precursor ions as assigned by Mascot. (A) Sample spectrum assigned to the peptide acetylated at the lysine with a Mascot score value of 87. (B) Spectrum of acetylated synthetic control peptide. (C) Sample spectrum assigned to the non-acetylated peptide with a Mascot score value of 65. (D) Spectrum of non-acetylated synthetic control peptide. (TIF) [file pone.0061867.s001.tif]

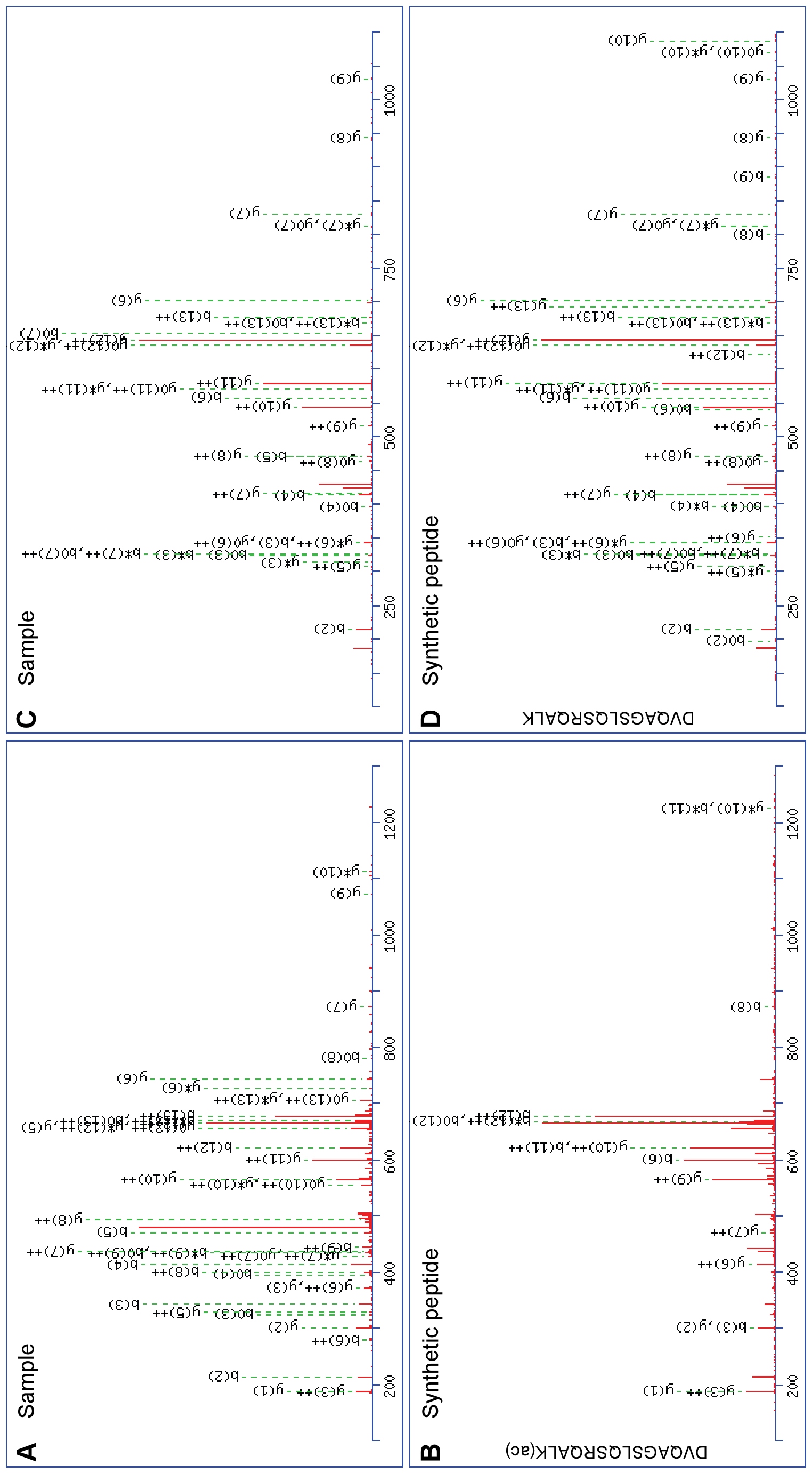

Supplement: Figure S2 — Evidence for acetylation of TCF4 at K150. Comparison of the MS/MS spectra for peptides found in the AspN-digested TCF4 sample and assigned by MASCOT (A, C) versus the MS/MS spectra of acetylated and non-acetylated versions of the synthetic peptide DVQAGSLQSRQALK (B, D). The pictures show the MS/MS spectra of the triply charged precursor ions as assigned by Mascot. (A) Sample spectrum assigned to the peptide acetylated at the lysine with a Mascot score value of 40. (B) Spectrum of acetylated synthetic control peptide. (C) Sample spectrum assigned to the non-acetylated peptide with a Mascot score value of 26, which is below the Mascot threshold value for indication of identity at p<0.05. (D) Spectrum of non-acetylated synthetic control peptide. (TIF) [file pone.0061867.s002.tif]

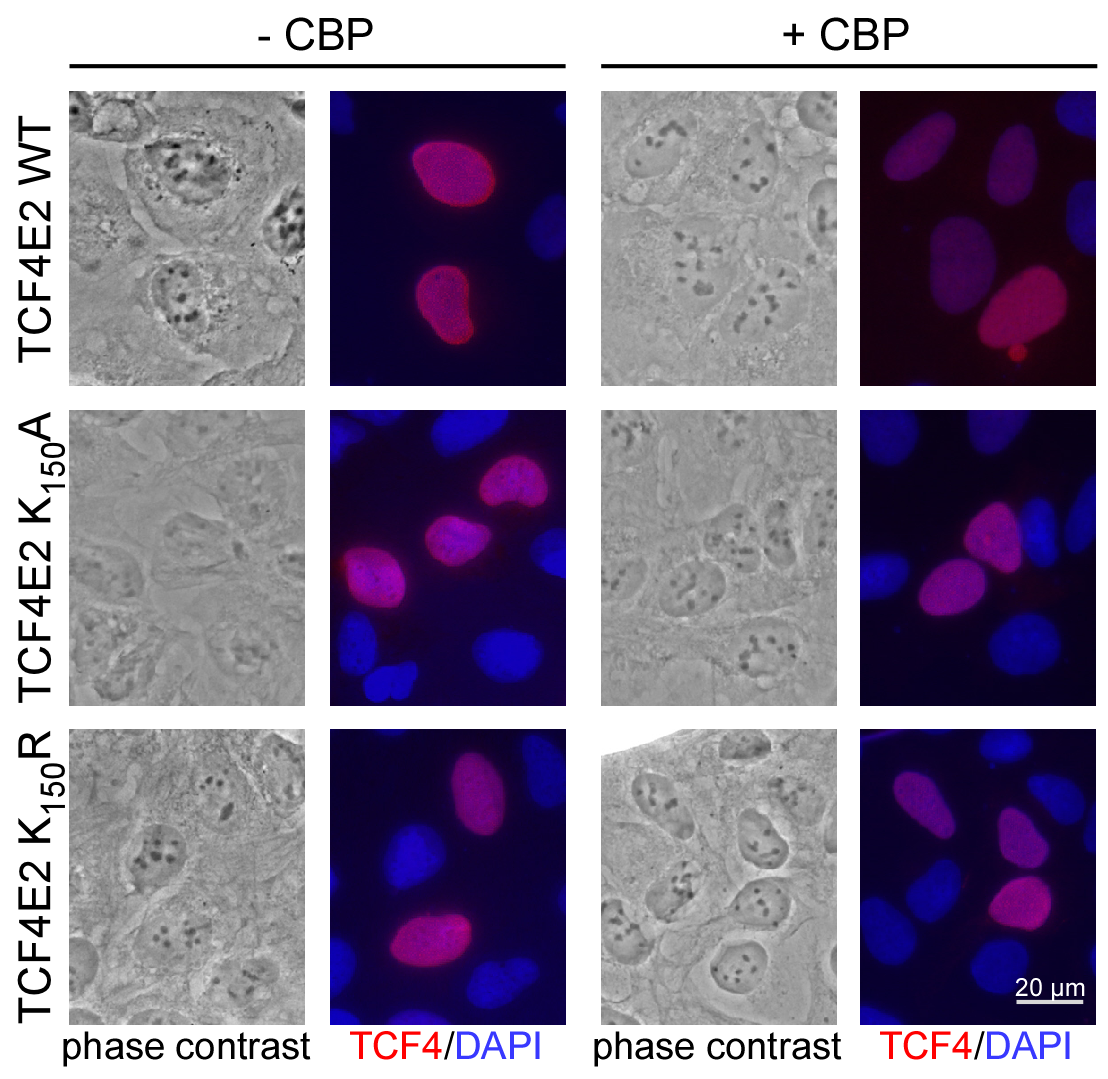

Supplement: Figure S3 — Intracellular localization of TCF4E2 WT, K150A and K150R. U-2 OS cells transfected with expression constructs for the TCF4E2 variants with or without CBP were stained with anti-HA antibodies and secondary antibodies coupled to Alexa-555 to visualize the TCF4E2 variants (TCF4, red). Nuclei were counterstained with DAPI. Phase contrast and overlay of TCF4E2 and DAPI staining are shown. Bar: 20 µm. (TIF) [file pone.0061867.s003.tif]

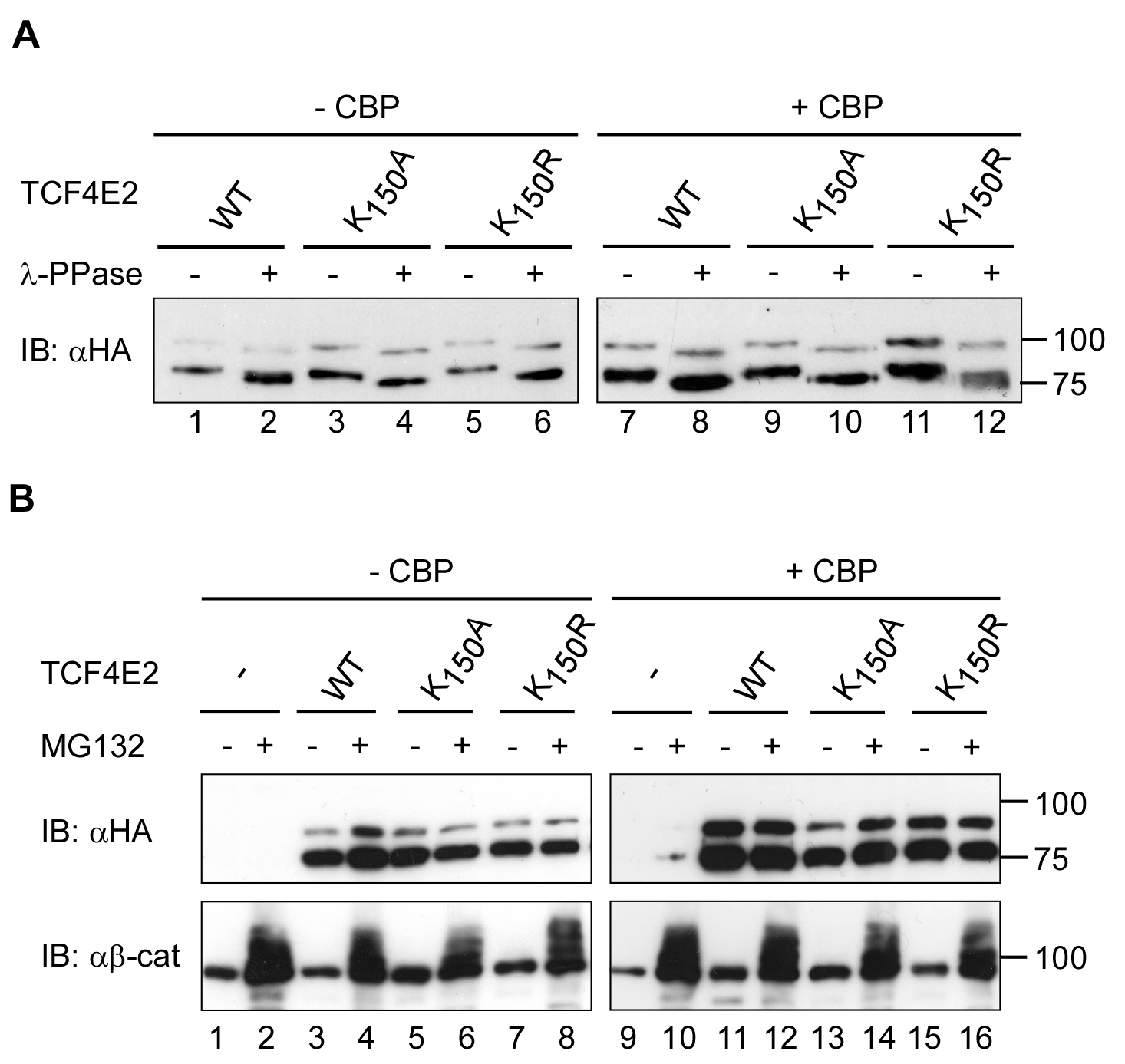

Supplement: Figure S4 — Acetylation of K150 does not affect phosphorylation or proteasomal degradation of TCF4E2. (A) To analyze if K150 acetylation influences the phosphorylation of TCF4E2, extracts of HEK293 cells transfected with the expression constructs for TCF4E2, TCF4E2K150A or TCF4E2K150R in the absence or presence of CBP were treated with λ-phosphatase (λ-PPase) and analyzed by SDS-PAGE and western blotting with anti-HA antibodies. Prior to λ-phosphatase treatment, all TCF4 variants showed comparable migration patterns by SDS-PAGE, suggesting that they are equally phosphorylated. λ-phosphatase treatment resulted in a mobility shift and faster migration of the TCF4 variants due to dephosphorylation. Again, wild-type TCF4E2 and TCF4E2 mutants showed uniform behavior. (B) HEK293 cells transfected with expression constructs for TCF4E2, TCF4E2K150A or TCF4E2K150R with or without CBP were treated with 20 µM MG132 for two hours prior to cell lysis and whole cell extracts were analyzed by SDS-PAGE and western blotting with anti-HA and anti-β-catenin (αβ-cat) antibodies. For TCF4E2 no change in protein amount and no additional protein bands that would indicate polyubiquitination were detected. The presence or absence of CBP made no difference. In contrast, for β-catenin, MG132 treatment resulted in the appearance of additional protein bands and a stronger signal, suggesting polyubiquitination of the protein. Molecular weight standards are indicated on the right of the panels. (TIF) [file pone.0061867.s004.tif]

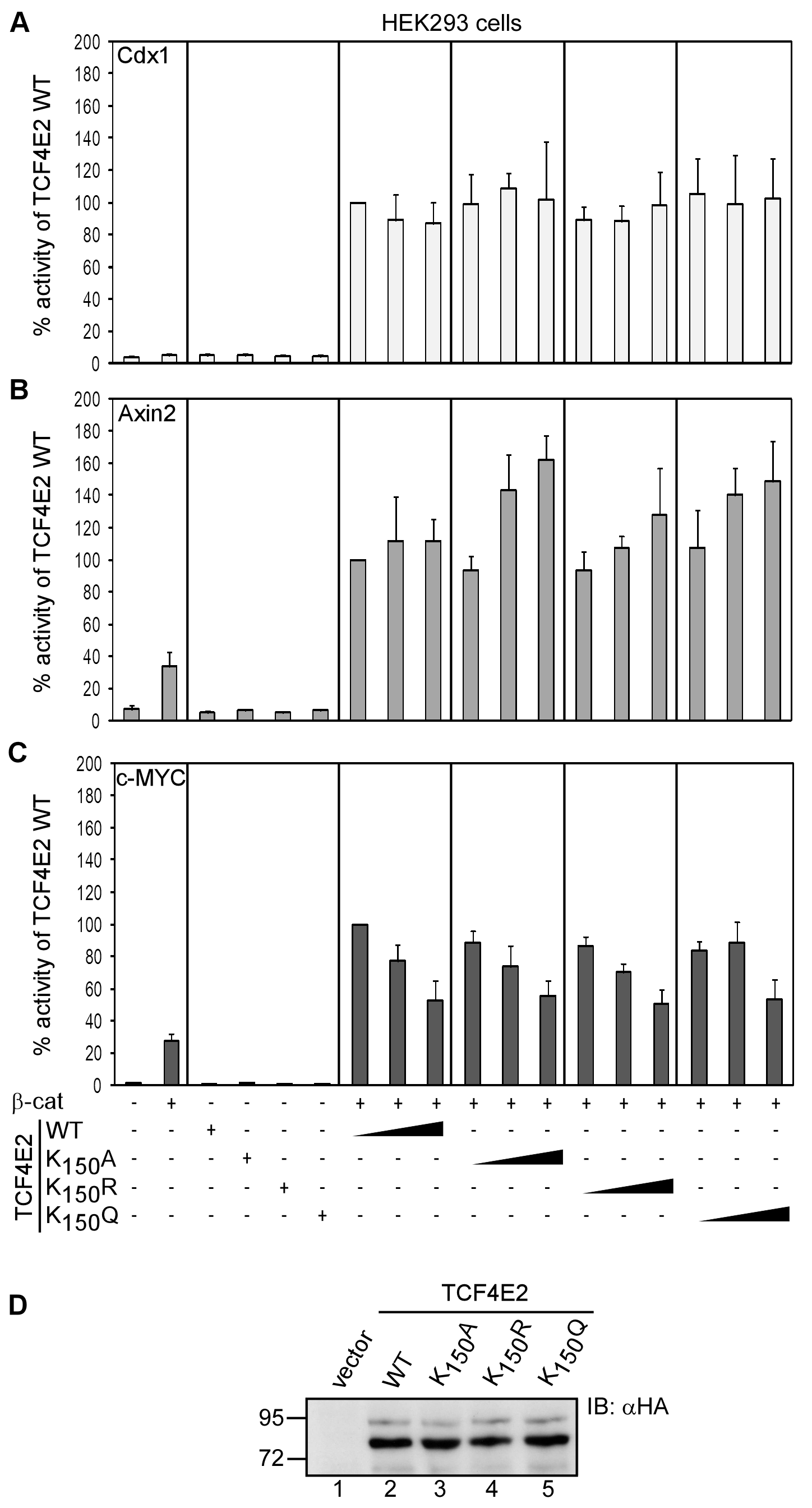

Supplement: Figure S5 — Mutation of K150 has no influence on the transactivation capacity of TCF4E2 at different promoters in HEK293 cells. HEK293 cells were cotransfected with combinations of firefly and Renilla luciferase reporter genes, control vector, expression vector for a constitutively active form of β-catenin and increasing amounts of TCF4E2 variants as indicated. Firefly expression was driven by promoters from the Wnt/β-catenin target genes Cdx1 (A), Axin2 (B) and c-MYC (C). Reporter gene activities were determined 40 h post transfection. Bars represent relative luciferase activity compared to values obtained with the lowest amount of TCF4E2 WT expression vector and β-catenin (set to 100%). The average values and standard deviations from at least three independent experiments are shown. (D) Expression levels of all TCF4E2 variants in whole cell extracts of cells transfected with 150 ng DNA as used in the luciferase reporter assays was controlled by SDS-PAGE and western blotting using anti-HA antibodies. Molecular weight standards are shown on the left. (TIF) [file pone.0061867.s005.tif]

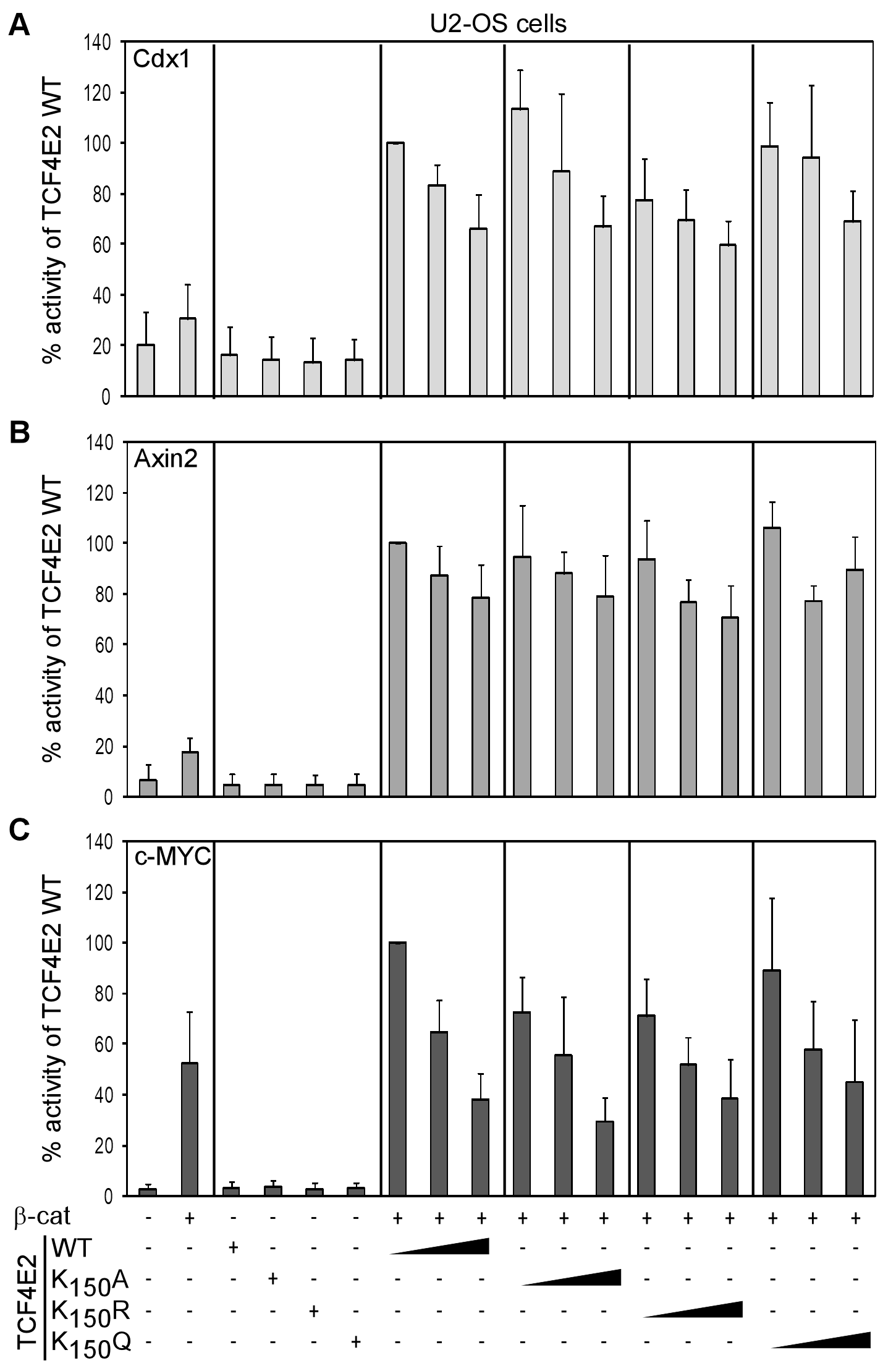

Supplement: Figure S6 — Mutation of K150 has no influence on the transactivation capacity of TCF4E2 at different promoters in U-2 OS cells. U-2 OS cells were cotransfected with combinations of firefly and Renilla luciferase reporter genes, control vector, expression vector for a constitutively active form of β-catenin and increasing amounts of TCF4E2 variants as indicated. Firefly expression was driven by promoters from the Wnt/β-catenin target genes Cdx1 (A), Axin2 (B) and c-MYC (C). Reporter gene activities were determined 40 h post transfection. Bars represent relative luciferase activity compared to values obtained with the lowest amount of TCF4E2 WT expression vector and β-catenin (set to 100%). The average values and standard deviations from at least three independent experiments are shown. (TIF) [file pone.0061867.s006.tif]
